# Completions and algebraic formulas for the coefficients of Ramanujan’s mock theta functions

**DOI:** 10.1007/s11139-020-00324-4

**Published:** 2020-10-20

**Authors:** David Klein, Jennifer Kupka

**Affiliations:** Fachbereich Mathematik, Technische Universität Darmstadt, Schlossgartenstrasse 7, 64289 Darmstadt, Germany

**Keywords:** Mock theta function, Harmonic weak Maass form, Theta lift, Traces of singular moduli, 11F37, 11F27

## Abstract

We present completions of mock theta functions to harmonic weak Maass forms of weight $\nicefrac {1}{2}$ and algebraic formulas for the coefficients of mock theta functions. We give several harmonic weak Maass forms of weight $\nicefrac {1}{2}$ that have mock theta functions as their holomorphic part. Using these harmonic weak Maass forms and the Millson theta lift, we compute finite algebraic formulas for the coefficients of the appearing mock theta functions in terms of traces of singular moduli.

## Introduction

Mock theta functions first appeared in Ramanujan’s last letter to his friend Hardy in 1920. In this letter he told Hardy that he had discovered a new class of functions which he called mock theta functions. Ramanujan did not give any definition of what a mock theta function should be, but listed 17 examples, divided into four groups of orders 3, 5, 7 and 10, respectively, given as *q*-hypergeometric series, and stated various identities between them and some analytical properties. For example, the four mock theta functions of order 3 that Ramanujan defined in his letter are

$$\begin{aligned} f(q)&:= \sum _{n=0}^{\infty } \frac{q^{n^{2}}}{\left( -q;q \right) _{n}^{2}}, \ \ \ \ \ \ \ \ \ \ \ \phi (q) := \sum _{n=0}^{\infty } \frac{q^{n^{2}}}{\left( -q^{2};q^{2} \right) _{n}}, \\ \psi (q)&:= \sum _{n=1}^{\infty } \frac{q^{n^{2}}}{\left( q;q^{2} \right) _{n}}, \ \ \ \ \ \ \ \ \ \ \ \ \chi (q) := \sum _{n=0}^{\infty } \frac{q^{n^{2}} \left( -q;q \right) _{n}}{\left( -q^{3};q^{3} \right) _{n}}, \end{aligned}$$

where we have used the standard notation

$$\begin{aligned} \left( a;q^{k} \right) _{n} := \prod _{m=0}^{n-1} \left( 1-a q^{mk} \right) . \end{aligned}$$

Since then many mathematicians (especially Watson in his work [17]) have dealt with Ramanujan’s 17 functions, and have proven many of the identities he had given. A number of 16 further mock theta functions were later found in Ramanujan’s Lost Notebook (see, e.g., [5, 15]), including seven functions of order 6. Other mathematicians have also discovered more mock theta functions that had not been considered before: In [9] Gordon and McIntosh found functions of order 8 while McIntosh also studied mock theta functions of order 2 in [13].

Articles that offer a good first overview on this topic are, for example, [8, 18]. A more detailed survey over all mock theta functions of the different orders, including their definitions, relations and transformation formulas is provided in [10]. In this paper we will use the standard definitions of the mock theta functions as given in [10].

One major breakthrough in a deeper understanding of mock theta functions came in 2002 when Sander Zwegers found a connection between mock theta functions and harmonic weak Maass forms of weight $\nicefrac {1}{2}$. He proved that a mock theta function could be completed to a harmonic weak Maass form of weight $\nicefrac {1}{2}$ by multiplying it by a suitable power of *q* and subsequently adding a certain non-holomorphic function to it. Zwegers considered these completions for the fifth- and seventh-order mock theta functions in his PhD thesis [20], and for two of the third-order mock theta functions in [19]. Moore followed the work of Zwegers and found transformation laws for mock theta functions of order 10 and their relation to harmonic weak Maass forms in [14]. Though Ramanujan had not explained what the order of a mock theta function should be, it turned out that the order is related to the level of the corresponding Maass form.

We will present such completions to a harmonic weak Mass form of weight $\nicefrac {1}{2}$ for 22 different mock theta functions of orders 2, 3, 6 and 8. For example, we will show for the sixth-order mock theta function

$$\begin{aligned} \sigma (q) := \sum _{n=0}^{\infty } \frac{q^{\frac{1}{2} (n+1) (n+2)} \left( -q;q \right) _{n}}{\left( q;q^{2} \right) _{n+1}} \end{aligned}$$

that the function $q^{-\frac{1}{12}} \ \sigma (q)$ is the holomorphic part of a harmonic weak Maass form of weight $\nicefrac {1}{2}$ for the subgroup

$$\begin{aligned} \{(\gamma ,\phi ) \in \text {Mp}_{2}(\mathbb {Z}) \ | \ \gamma \in \Gamma (6) \} \end{aligned}$$

of the metaplectic group $\text {Mp}_{2}(\mathbb {Z})$, where $\Gamma (6)$ is the principal congruence subgroup of level 6.

A further example of what we will prove is that, if

$$\begin{aligned} F(\tau ) = \left( \begin{array}{c} f_{0}(\tau ) \\ f_{1}(\tau ) \\ f_{2}(\tau ) \\ f_{3}(\tau ) \\ f_{4}(\tau ) \\ f_{5}(\tau ) \end{array}\right) := \left( \begin{array}{c} \sqrt{8} \ q^{-\frac{1}{12}} \ \sigma (q) \\ 2 \ q^{\frac{1}{4}} \ \rho (q) \\ q^{-\frac{1}{48}} \ \phi (q^{\frac{1}{2}}) \\ q^{-\frac{1}{48}} \ \phi (-q^{\frac{1}{2}}) \\ \sqrt{2} \ q^{-\frac{3}{16}} \ \psi (q^{\frac{1}{2}}) \\ \sqrt{2} \ q^{-\frac{3}{16}} \ \psi (-q^{\frac{1}{2}}) \end{array}\right) \end{aligned}$$

with $q:=e^{2 \pi i \tau }$, $\tau \in \mathbb {H}$, and the mock theta functions $\sigma$, $\rho$, $\phi$ and $\psi$ of order 6, then the function

$$\begin{aligned} \widetilde{F}(\tau ) :=&\sqrt{2} \ f_{0}(\tau ) \ [-(\mathfrak {e}_{2}-\mathfrak {e}_{22})-(\mathfrak {e}_{10}-\mathfrak {e}_{14})] + 2 \ f_{1}(\tau ) \ [-(\mathfrak {e}_{6}-\mathfrak {e}_{18})] \\&+ (f_{2}(\tau )+f_{3}(\tau )) \ [(\mathfrak {e}_{1}-\mathfrak {e}_{23})-(\mathfrak {e}_{7}-\mathfrak {e}_{17})] \\&+ (f_{2}(\tau )-f_{3}(\tau )) \ [(\mathfrak {e}_{5}-\mathfrak {e}_{19})-(\mathfrak {e}_{11}-\mathfrak {e}_{13})] \\&+ \sqrt{2} \ (f_{4}(\tau )+f_{5}(\tau )) \ (\mathfrak {e}_{3}-\mathfrak {e}_{21}) + \sqrt{2} \ (f_{4}(\tau )-f_{5}(\tau )) \ [-(\mathfrak {e}_{9}-\mathfrak {e}_{15})], \end{aligned}$$

where $\mathfrak {e}_r$ are the standard basis vectors of the group algebra $\mathbb {C}[\mathbb {Z}/24\mathbb {Z}]$, is the holomorphic part of a harmonic weak Maass form of weight $\nicefrac {1}{2}$ for the dual Weil representation. This result opens up the possibility to use the powerful tool of theta lifts between spaces of modular forms.

The Millson theta lift, which maps weight 0 to weight $\nicefrac {1}{2}$ harmonic weak Maass forms, uses the Millson theta function as an integration kernel and was studied in great detail by Alfes in her thesis [1] and by Alfes-Neumann and Schwagenscheidt [2]. In particular, Alfes-Neumann found formulas for the coefficients of the holomorphic part of the Millson theta lift in terms of traces of singular moduli. By writing the harmonic weak Maass form of weight $\nicefrac {1}{2}$ containing the mock theta functions as the Millson theta lift of a suitable weakly holomorphic modular form, we can derive finite algebraic formulas for the coefficients of the considered mock theta functions in terms of traces of singular moduli. Continuing our example from above, we will prove that the coefficients $a_\sigma (n)$ of the mock theta function $\sigma$ of order 6 are given by

$$\begin{aligned} a_\sigma (n)=-\frac{i}{4\sqrt{48n-4}}\big (tr ^+_{e_{(6),1}}(4-48n,2)-tr ^-_{e_{(6),1}}(4-48n,2)\big ), \end{aligned}$$

where the trace functions $tr ^+_{e_{(6),1}}$ and $tr ^-_{e_{(6),1}}$ are given as in (2.4), and $e_{(6),1}\in M^!_0(12)$ is defined as

$$\begin{aligned} e_{(6),1}(z):=\bigg (\frac{\eta (z)\eta (3z)}{\eta (4z)\eta (12z)}\bigg )^2-16\bigg (\frac{\eta (4z)\eta (12z)}{\eta (z)\eta (3z)}\bigg )^2 \end{aligned}$$

with $\eta (\tau ) = q^{\frac{1}{24}} \ \prod _{n=1}^{\infty } (1-q^{n})$ denoting the Dedekind eta function. Similar formulas for the order 3 mock theta functions *f* and $\omega$ (see, e.g., [7] for its definition) have already been proven by Bruinier and Schwagenscheidt in [7].

This paper is organized as follows. We will start with the necessary definitions, notations and results in Sect. 2, followed by the results on the completions and formulas for the coefficients of the mock theta functions in Sect. 3. We will consider mock theta functions of different orders separately and in Sect. 3.1, those of order 6, will be worked out in detail. As the ideas and strategies for the other orders are very similar to the case of order 6, the subsections corresponding to the other orders only contain known results and no proofs.

Most of the results presented in this paper first appeared in our Master’s theses [12] and [11] where they also have been proven in more detail.

## Preliminaries

### Lattices, the Weil representation and theta functions

Let $N>0$ be an integer. We consider the lattice $L=\mathbb {Z}$ with the quadratic form $n \mapsto N n^{2}$. The discriminant group $\mathcal {D}:=L'/L$ can then be identified with $\mathbb {Z}/2N\mathbb {Z}$ together with the $\mathbb {Q}/\mathbb {Z}$-valued quadratic form $r\mapsto \frac{r^2}{4N} \ (\text {mod} \ \mathbb {Z})$. The associated bilinear form on $\mathcal {D}$ is $(r,r')=\frac{rr'}{2N} \ (\text {mod} \ \mathbb {Z})$.

For $r\in L'/L$ we define $\mathfrak {e}_r$ to be the standard basis vectors of the group algebra $\mathbb {C}[L'/L]$ equipped with the standard inner product $\langle \cdot ,\cdot \rangle$ satisfying $\langle \mathfrak {e}_r,\mathfrak {e}_{r'}\rangle =\delta _{r,r'}$. The associated *Weil representation* $\rho _L$ is defined on the generators $$T=\big (({\begin{matrix}1&{}1\\ 0&{}1 \end{matrix}}),1 \big )$$ and $$S=\big (({\begin{matrix}0&{}-1\\ 1&{}0 \end{matrix}}),\sqrt{\tau } \big )$$ of the metaplectic group $\text {Mp}_{2}(\mathbb {Z})$ by

2.1 $$\begin{aligned} \rho _L(T)\mathfrak {e}_r=e\big (Q(r)\big )\mathfrak {e}_r\ \ \text { and }\ \ \rho _L(S)\mathfrak {e}_r=\frac{e(-1/8)}{\sqrt{2N}}\sum _{r'(2N)}e\big (-(r,r')\big )\mathfrak {e}_{r'}, \end{aligned}$$

where $e(z)=e^{2\pi iz}$ for $z\in \mathbb {C}$ and $\sqrt{z}=z^{\frac{1}{2}}$ always denotes the principal branch of the square root. The dual Weil representation corresponds to the lattice *L* with quadratic form $-Q$ and will be denoted by $\overline{\rho }_L$.

Let *N* be as above and $a \in \mathbb {Z}$. For $\tau \in \mathbb {H}$ we define the *unary theta function* $\theta _{N}$ *of level* *N* as

$$\begin{aligned} \theta _{N}(\tau ) := \sum _{a \ (2N)} \theta _{N,a} (\tau ) \ \mathfrak {e}_{a}, \ \ \text { where } \ \ \theta _{N,a}(\tau ) := \sum _{n \equiv a \ (2N)} n \ q^{\frac{n^{2}}{4N}} = \sum _{n \equiv a \ (2N)} n \ e^{2 \pi i \tau \frac{n^{2}}{4N}}. \end{aligned}$$

The definition of $\theta _{N,a}$ depends only on $a \ (2N)$. If we consider the lattice above as well as its associated Weil representation, then the vector valued theta function $\theta _{N}$ is a holomorphic vector valued modular form of weight $\nicefrac {3}{2}$ for this Weil representation. Thus, the function $\theta _{N,a}$ is holomorphic on $\mathbb {H}$ and has the modular transformation properties

2.2 $$\begin{aligned} \theta _{N,a}(\tau +1) = e \left( \frac{a^{2}}{4N} \right) \ \theta _{N,a}(\tau ) \end{aligned}$$

and

2.3 $$\begin{aligned} \theta _{N,a} \left( -\frac{1}{\tau } \right) = \tau ^{\frac{3}{2}} \ \frac{e \left( -\frac{1}{8} \right) }{\sqrt{2N}} \ \sum _{k \ (2N)} e \left( -\frac{ak}{2N} \right) \ \theta _{N,k}(\tau ). \end{aligned}$$

Let *Q* be an exact divisor of *N*, i.e. $Q\in \mathbb {Z}_{>0}$ with $Q\vert N$ and $\gcd (N/Q,Q)=1$. The *Atkin–Lehner involution* associated to *Q* is then defined by any matrix

$$\begin{aligned} W_Q^N=\begin{pmatrix} Q\alpha &{}\beta \\ N\gamma &{}Q\delta \end{pmatrix}, \end{aligned}$$

where $\alpha ,\beta ,\gamma ,\delta \in \mathbb {Z}$ with $\det (W_Q^N)=Q$. The map

$$\begin{aligned} W_Q^N:\ M_k(N)\rightarrow M_k(N),\ \ f\mapsto f|_kW_Q^N \end{aligned}$$

does not depend on the choice of $\alpha ,\beta ,\gamma$ and $\delta$ and defines an involution. For two exact divisors $Q,Q'$ of *N* we define the product

$$\begin{aligned} Q*Q':=\frac{Q\cdot Q'}{\gcd (Q,Q')^2}, \end{aligned}$$

which is compatible with the action of the Petersson slash operator, i.e. we have

$$\begin{aligned} f|_kW_{Q*Q'}^N=f|_kW_Q^N|_kW_{Q'}^N. \end{aligned}$$

The automorphism group $\text {Aut}(\mathbb {Z}/2N\mathbb {Z})$ acts on vector valued modular forms $f=\sum _{r\in \mathbb {Z}/2N\mathbb {Z}}f_r \ \mathfrak {e}_r$ for $\rho _L$ or $\overline{\rho }_L$ by

$$\begin{aligned} f^\sigma =\sum _{r}f_r \ \mathfrak {e}_{\sigma (r)}. \end{aligned}$$

These automorphisms are all involutions, which are also called Atkin–Lehner involutions and correspond to exact divisors *Q* of *N*. The automorphism $\sigma _Q$ corresponding to *Q* is defined by the two equations

$$\begin{aligned} \sigma _Q(r)\equiv -r \ (2Q)\ \text {and}\ \sigma _Q(r)\equiv r \ (2N/Q) \end{aligned}$$

for an element $r\in \mathbb {Z}/2N\mathbb {Z}$.

### Harmonic Maass forms and the $\xi$-operator

Vector valued harmonic weak Maass forms were first introduced by Bruinier and Funke [6]. We will consider a more general setting than they have in their article.

Let *V* be a vector space over $\mathbb {C}$ of finite dimension *d* and let $k \in \frac{1}{2} \mathbb {Z}$ with $k \ne 1$. For $\tau \in \mathbb {H}$ we put $u:=\text {Re}(\tau )$ and $v:=\text {Im}(\tau )$, so that $\tau =u+iv$. Moreover, recall the weight *k* hyperbolic Laplace operator, given by

$$\begin{aligned} \Delta _{k} = -v^{2} \ \left( \frac{\partial ^{2}}{\partial u^{2}} + \frac{\partial ^{2}}{\partial v^{2}} \right) + ikv \ \left( \frac{\partial }{\partial u} + i \ \frac{\partial }{\partial v} \right) . \end{aligned}$$

Let $\rho : \text {Mp}_{2}(\mathbb {Z}) \rightarrow \text {GL}(V)$ be a unitary representation of $\text {Mp}_{2}(\mathbb {Z})$ that satisfies $\rho (T)^{N}=\text {Id}$ for some $N \in \mathbb {N}$, let $f: \mathbb {H} \rightarrow V$ be a twice continuously differentiable function and $\Gamma \subseteq \text {Mp}_{2}(\mathbb {Z})$ a subgroup of finite index. We call *f* a *harmonic weak Maass form of weight* *k* with respect to the representation $\rho$ and the group $\Gamma$ if

1. $$f(\gamma \tau )=\phi (\tau )^{2k} \ \rho (\gamma ,\phi ) \ f(\tau )$$ for all $$(\gamma ,\phi ) \in \Gamma $$,
2. there is a constant $C>0$ such that for any cusp $s \in \mathbb {Q} \cup \{\infty \}$ of $\Gamma$ and $(\delta ,\phi ) \in \text {Mp}_{2}(\mathbb {Z}$) with $\delta \infty =s$ the function $f_{s}(\tau ):=\phi (\tau )^{-2k} \ \rho ^{-1}(\delta ,\phi ) \ f(\delta \tau )$ satisfies $f_{s}(\tau )=O(e^{Cv})$ as $v \rightarrow \infty$ (uniformly in *u*),
3. $$\Delta _{k} f=0$$.

Condition (ii) says that *f* increases at most linear exponentially at all cusps of $\Gamma$.

The space of these forms is denoted by $H_{k,\rho }(\Gamma )$. If we have $\Gamma =\text {Mp}_{2}(\mathbb {Z})$, we write as an abbreviation $H_{k,\rho }(\text {Mp}_{2}(\mathbb {Z}))=:H_{k,\rho }$. Further, let $M^{!}_{k,\rho }$ be its subspace of weakly holomorphic modular forms, consisting of those forms in $H_{k,\rho }$ that are holomorphic on $\mathbb {H}$.

A harmonic weak Maass form $f \in H_{k,\rho }$ has a unique decomposition $f = f^{+}+f^{-}$, where $f^{+}$ is the *holomorphic part* and $f^{-}$ is the *non-holomorphic part of* *f*. If we write the Fourier expansion of the holomorphic part of $f \in H_{k,\rho }$ as

$$\begin{aligned} f^{+}(\tau ) = \sum _{n \in \mathbb {Z}} a^{+}(n) \ e \left( \frac{n \tau }{N} \right) , \end{aligned}$$

where $a^{+}(n)$ are vector valued coefficients, then the Fourier polynomial

$$\begin{aligned} P(f)(\tau ) = \sum _{n \in \mathbb {Z}, n \le 0} a^{+}(n) \ e \left( \frac{n\tau }{N} \right) \end{aligned}$$

is called the *principal part of* *f*.

For $f \in H_{k,\rho }$ the differential operator $\xi _{k}$ is given by

$$\begin{aligned} \xi _{k}(f)(\tau ) = 2i \ v^{k} \ \overline{\frac{\partial }{\partial \overline{\tau }} f(\tau )}. \end{aligned}$$

The operator $\xi _{k}$ is antilinear and defines a surjective mapping $\xi _{k}: H_{k,\rho } \rightarrow M_{2-k,\overline{\rho }}^{!}$ with kernel given by $M_{k,\rho }^{!}$. We can use $\xi _{k}$ to define the subspace

$$\begin{aligned} H_{k,\rho }^{+} := \{f \in H_{k,\rho } \ | \ \xi _{k}(f) \in S_{2-k,\overline{\rho }} \}, \end{aligned}$$

so that $H_{k,\rho }^{+}$ consists of all harmonic weak Maass forms in $H_{k,\rho }$ that are mapped to cusp forms under $\xi _{k}$. The holomorphic part $f^{+}$ of $f \in H_{k,\rho }^{+}$ is sometimes also called a *mock modular form*, and $\xi _{k}f$ is called the *shadow* of *f*.

We will use the following lemma when we prove our formulas for the coefficients.

#### Lemma 2.1

[7, Lemma 2.3]. Let *G* be a harmonic weak Maass form of weight $2-k\in \nicefrac {1}{2}+\mathbb {Z}$ for $\rho _L$ or $\overline{\rho }_L$ whose principal part vanishes and which maps to a cusp form under $\xi _{2-k}$ (or a holomorphic modular form if $k=\nicefrac {1}{2}$). Then *G* is a cusp form.

### The Millson theta lift and traces of CM-values

For a discriminant $D<0$ and $r\in \mathbb {Z}$ with $D\equiv r^2 \ (4N)$ denote by $\mathcal {Q}_{N,D,r}$ the set of integral binary quadratic forms $Q(x,y)=ax^2+bxy+cy^2$ of discriminant $D=b^2-4ac$ and satisfying $N\vert a$ and $b\equiv r \ (2N)$. This set splits into the sets of positive and negative definite quadratic forms, which we denote by $\mathcal {Q}^+_{N,D,r}$ and $\mathcal {Q}^-_{N,D,r}$, respectively. The group $\Gamma _0(N)$ acts on both of these sets with finitely many orbits and the number $\omega _Q=\frac{1}{2}\vert \Gamma _0(N)_Q\vert$ is finite. For each $Q\in \mathcal {Q}^+_{N,D,r}$ the equation $Q(z_Q,1)=0$ is solved by the associated *CM-point* $z_Q=(-b+i\sqrt{\vert D\vert })/2a$.

For a weakly holomorphic modular form $F\in M_0^!(N)$ of weight 0 for $\Gamma _0(N)$ we define the two *trace functions*

2.4 $$\begin{aligned} \text {tr}_F^+(D,r)=\sum _{Q\in \mathcal {Q}^+_{N,D,r}/\Gamma _0(N)}\frac{F(z_Q)}{\omega _Q}\ \ \text { and }\ \ \text {tr}_F^-(D,r)=\sum _{Q\in \mathcal {Q}^-_{N,D,r}/\Gamma _0(N)}\frac{F(z_Q)}{\omega _Q}. \end{aligned}$$

The *Millson theta lift* $\mathcal {I}^M(F,\tau )$ of a weakly holomorphic modular form $F\in M_0^!(N)$ is defined as an integral

$$\begin{aligned} \mathcal {I}^M(F,\tau )=\frac{i}{\sqrt{N}}\int _{\Gamma _0(N)\setminus \mathbb {H}}F(z)\ \Theta _M(\tau ,z)\ \frac{\mathrm{d}x\mathrm{d}y}{y^2}, \end{aligned}$$

where we write $z=x+iy$ and $\Theta _M(\tau ,z)$ denotes the Millson theta function. The theta function $\Theta _M(\tau ,z)$ is $\Gamma _0(N)$-invariant in the variable *z* and transforms like a modular form of weight $\nicefrac {1}{2}$ for the dual Weil representation $\overline{\rho }_L$ in the variable $\tau$. The assignment $F\mapsto \mathcal {I}^M(F,\tau )$ then defines a map $\mathcal {I}^M:\ M_0^!(N)\rightarrow H_{1/2,\overline{\rho }_L}$. For more details see [1] or [2]. As it turns out, the coefficients of the holomorphic part of the Millson theta lift can be computed using the trace functions which we defined above.

#### Theorem 2.2

[1, Theorem 4.3.1] Let $F\in H_0^+(N)$ be a harmonic weak Maass form of weight 0 for $\Gamma _0(N)$, $D<0$ a discriminant and $r\in L'/L$ with $D\equiv r^2 \ (4N)$. Then the coefficient of index $(-D,r)$ of the holomorphic part of the Millson theta lift $\mathcal {I}^M(\tau ,F)$ is given by

$$\begin{aligned} \frac{i}{\sqrt{-D}}\big (tr _F^+(D,r)-tr _F^-(D,r)\big ). \end{aligned}$$

## Completions and algebraic formulas for the coefficients of mock theta functions

### Mock theta functions of order 6

We want to complete sixth-order mock theta functions to harmonic weak Maass forms and want to derive algebraic formulas for their coefficients. For this aim we will first construct two different vector valued Maass forms, one containing the sixth-order functions $\sigma , \rho , \phi$ and $\psi$ and the other comprising $\mu , \lambda , \nu$ and $\xi$. Their definitions, and also the definitions of the mock theta functions of other orders, can be found in [10]. Afterwards we will derive the transformation behaviour of its components. Starting from our vectors we will further construct two vector valued harmonic weak Maass forms for the dual Weil representation. We will then be able to obtain algebraic formulas for the coefficients of the mentioned mock theta functions.

#### Definition 3.1

For $\tau \in \mathbb {H}$ we define the vector valued functions

$$\begin{aligned} F_{(6),1}(\tau ) := \left( \begin{array}{c} \sqrt{8} \ q^{-\frac{1}{12}} \ \sigma (q) \\ 2 \ q^{\frac{1}{4}} \ \rho (q) \\ q^{-\frac{1}{48}} \ \phi (q^{\frac{1}{2}}) \\ q^{-\frac{1}{48}} \ \phi (-q^{\frac{1}{2}}) \\ \sqrt{2} \ q^{-\frac{3}{16}} \ \psi (q^{\frac{1}{2}}) \\ \sqrt{2} \ q^{-\frac{3}{16}} \ \psi (-q^{\frac{1}{2}}) \end{array}\right) \ \ \ \ \ \text {and} \ \ \ \ \ F_{(6),2}(\tau ) := \left( \begin{array}{c} -\sqrt{2} \ q^{-\frac{1}{12}} \ \mu (q) \\ - q^{\frac{1}{4}} \ \lambda (q) \\ -2 \ q^{-\frac{1}{48}} \ \nu (q^{\frac{1}{2}}) \\ -2 \ q^{-\frac{1}{48}} \ \nu (-q^{\frac{1}{2}}) \\ -\sqrt{8} \ q^{-\frac{3}{16}} \ \xi (q^{\frac{1}{2}}) \\ -\sqrt{8} \ q^{-\frac{3}{16}} \ \xi (-q^{\frac{1}{2}}) \end{array}\right) \end{aligned}$$

with $$q=e^{2 \pi i \tau }$$.

These two functions have the same modular transformation properties as the following lemma states.

#### Lemma 3.2

For $j=1,2$ and $\tau \in \mathbb {H}$ the function $F_{(6),j}$ satisfies

3.1 $$\begin{aligned} F_{(6),j}(\tau +1) = \left( \begin{matrix} \zeta _{12}^{-1} &{} 0 &{} 0 &{} 0 &{} 0 &{} 0 \\ 0 &{} i &{} 0 &{} 0 &{} 0 &{} 0 \\ 0 &{} 0 &{} 0 &{} \zeta _{48}^{-1} &{} 0 &{} 0 \\ 0 &{} 0 &{} \zeta _{48}^{-1} &{} 0 &{} 0 &{} 0 \\ 0 &{} 0 &{} 0 &{} 0 &{} 0 &{} \zeta _{16}^{-3} \\ 0 &{} 0 &{} 0 &{} 0 &{} \zeta _{16}^{-3} &{} 0 \end{matrix}\right) \ F_{(6),j}(\tau ) \end{aligned}$$

and

3.2 $$\begin{aligned} \frac{1}{\sqrt{-i \tau }} \ F_{(6),j} \left( -\frac{1}{\tau } \right) = \left( \begin{matrix} 0 &{} 0 &{} \frac{1}{\sqrt{3}} &{} 0 &{} \sqrt{\frac{2}{3}} &{} 0 \\ 0 &{} 0 &{} \sqrt{\frac{2}{3}} &{} 0 &{} -\frac{1}{\sqrt{3}} &{} 0 \\ \frac{1}{\sqrt{3}} &{} \sqrt{\frac{2}{3}} &{} 0 &{} 0 &{} 0 &{} 0 \\ 0 &{} 0 &{} 0 &{} \frac{1}{\sqrt{3}} &{} 0 &{} -\sqrt{\frac{2}{3}} \\ \sqrt{\frac{2}{3}} &{} -\frac{1}{\sqrt{3}} &{} 0 &{} 0 &{} 0 &{} 0 \\ 0 &{} 0 &{} 0 &{} -\sqrt{\frac{2}{3}} &{} 0 &{} -\frac{1}{\sqrt{3}} \end{matrix}\right) \ F_{(6),j}(\tau ) + R_{(6)}(\tau ), \end{aligned}$$

where

$$\begin{aligned} R_{(6)}(\tau ) := \frac{\sqrt{6}i}{\tau } \ \left( \begin{matrix} -\sqrt{8} \ J_{1}(\frac{6 \pi i}{\tau }) \\ -2 \ J(\frac{6 \pi i}{\tau }) \\ J_{1}(\frac{3 \pi i}{2 \tau }) \\ K_{1}(\frac{3 \pi i}{\tau }) \\ \frac{1}{\sqrt{2}} \ J(\frac{3 \pi i}{2 \tau }) \\ \sqrt{2} \ K(\frac{3 \pi i}{\tau }) \end{matrix}\right) , \end{aligned}$$

and $J, J_{1}, K, K_{1}$ are given by

$$\begin{aligned} J(\alpha ) \&= \ \int _{0}^{\infty } \frac{e^{-\alpha x^{2}}}{\cos h(\alpha x)} \ \mathrm{d}x, \ \ \ \ \ \ \ \ \ \ \ \ \ \ \ \ \ \ \ K(\alpha ) \ = \ \int _{0}^{\infty } e^{-\frac{1}{2} \alpha x^{2}} \ \frac{\cos h \left( \frac{1}{2} \alpha x \right) }{\cos h(\alpha x)} \ \mathrm{d}x, \\ J_{1}(\alpha ) \&= \ \int _{0}^{\infty } e^{-\alpha x^{2}} \ \frac{\cos h \left( \frac{2}{3} \alpha x \right) }{\cos h(\alpha x)} \ \mathrm{d}x, \ \ \ \ \ \ \ K_{1}(\alpha ) \ = \ \int _{0}^{\infty } e^{-\frac{1}{2} \alpha x^{2}} \ \frac{\cos h \left( \frac{5}{6} \alpha x \right) - \cos h \left( \frac{1}{6} \alpha x \right) }{\cos h(\alpha x)} \ \mathrm{d}x. \end{aligned}$$

#### Proof

Let $j=1$. The formula (3.1) follows directly if we insert $\tau +1$.

If we use the transformation formulas for $\sigma (q)$, $\rho (q)$, $\phi (-q)$ and $\psi (-q)$ in [10], p. 123 with $\alpha =\nicefrac {3 \pi i}{\tau }$ (which implies $q=e^{-\nicefrac {3 \pi i}{\tau }}$, $\beta =-\nicefrac {\pi i \tau }{3}$ and $q_{1}=e^{\nicefrac {2 \pi i \tau }{6}}$), as well as the formulas for $\phi (q)$ and $\psi (q)$ with $\alpha =\nicefrac {3 \pi i}{2 \tau }$ (which yields $q=e^{-\nicefrac {3 \pi i}{2 \tau }}$, $\beta =-\nicefrac {2 \pi i \tau }{3}$ and $q_{1}=e^{\nicefrac {2 \pi i \tau }{3}}$), we obtain (3.2).

For $j=2$ the proof is analogous, using the transformation formulas for $\mu$, $\lambda$, $\nu$ and $\xi$. $\square$

We can now write the function $R_{(6)}$ from the previous lemma in terms of integrals over sums of theta functions $\theta _{N,a}$ which have been defined in Sect. 2.1.

#### Lemma 3.3

For $\tau \in \mathbb {H}$ we have

3.3 $$\begin{aligned} R_{(6)}(\tau ) = \frac{i^{\frac{3}{2}}}{\sqrt{24}} \ \int _{0}^{i \infty } \frac{g_{(6)}(z)}{\sqrt{-i(z \tau -1)}} \ \mathrm{d}z, \end{aligned}$$

where $g_{(6)}$ is the vector $(g_{(6),0},g_{(6),1},g_{(6),2},g_{(6),3},g_{(6),4},g_{(6),5})^{T}$ and

$$\begin{aligned} g_{(6),0}(z)&:= \sqrt{2} \ (\theta _{12,2}(z)+\theta _{12,10}(z)), \\ g_{(6),1}(z)&:= 2 \ \theta _{12,6}(z), \\ g_{(6),2}(z)&:= -(\theta _{12,1}(z)+\theta _{12,5}(z)-\theta _{12,7}(z)-\theta _{12,11}(z)), \\ g_{(6),3}(z)&:= -(\theta _{12,1}(z)-\theta _{12,5}(z)-\theta _{12,7}(z)+\theta _{12,11}(z)), \\ g_{(6),4}(z)&:= -\sqrt{2} \ (\theta _{12,3}(z)-\theta _{12,9}(z)), \\ g_{(6),5}(z)&:= -\sqrt{2} \ (\theta _{12,3}(z)+\theta _{12,9}(z)). \end{aligned}$$

The integration over a vector valued function in the lemma means that we integrate each of its components.

#### Proof

Let

$$\begin{aligned} M_{(6)} := \left( \begin{matrix} 0 &{} 0 &{} \frac{1}{\sqrt{3}} &{} 0 &{} \sqrt{\frac{2}{3}} &{} 0 \\ 0 &{} 0 &{} \sqrt{\frac{2}{3}} &{} 0 &{} -\frac{1}{\sqrt{3}} &{} 0 \\ \frac{1}{\sqrt{3}} &{} \sqrt{\frac{2}{3}} &{} 0 &{} 0 &{} 0 &{} 0 \\ 0 &{} 0 &{} 0 &{} \frac{1}{\sqrt{3}} &{} 0 &{} -\sqrt{\frac{2}{3}} \\ \sqrt{\frac{2}{3}} &{} -\frac{1}{\sqrt{3}} &{} 0 &{} 0 &{} 0 &{} 0 \\ 0 &{} 0 &{} 0 &{} -\sqrt{\frac{2}{3}} &{} 0 &{} -\frac{1}{\sqrt{3}} \end{matrix}\right) . \end{aligned}$$

Replacing $\tau$ by $-\nicefrac {1}{\tau }$ in the transformation formula for *S* and subsequently multiplying both sides by $\frac{1}{\sqrt{-i \tau }} \ M_{(6)}$ yields

$$\begin{aligned} R_{(6)}(\tau )=-\frac{1}{\sqrt{-i \tau }} \ M_{(6)} \ R_{(6)} \left( -\frac{1}{\tau } \right) . \end{aligned}$$

If we choose $\tau :=it$ with $t \in \mathbb {R}$, $t>0$, we get

$$\begin{aligned} R_{(6)}(it) = -\frac{1}{\sqrt{t}} \ M_{(6)} \ R_{(6)} \left( \frac{i}{t} \right) . \end{aligned}$$

We consider the first component

$$\begin{aligned} \sqrt{6t} \ \left( -\frac{1}{\sqrt{3}} \ J_{1} \left( \frac{3 \pi t}{2} \right) - \frac{1}{\sqrt{3}} \ J \left( \frac{3 \pi t}{2} \right) \right) \end{aligned}$$

of this vector. If we use the identity $J_{1}(\alpha )=\frac{1}{2} \ J(\alpha ) + \frac{1}{6} \ J(\frac{\alpha }{9})$ (see, e.g., [10], p. 122), the partial fraction decomposition

$$\begin{aligned} \frac{1}{\cos h(\pi y)} = -\frac{i}{\pi } \ \sum _{n \in \mathbb {Z}} \frac{1}{y-i \left( 2n+\frac{1}{2} \right) }-\frac{i}{\pi } \ \sum _{n \in \mathbb {Z}} \frac{1}{-y-i \left( 2n+\frac{1}{2} \right) } \end{aligned}$$

and the identity

$$\begin{aligned} \int _{-\infty }^{\infty } \frac{e^{-\pi t y^{2}}}{y-ir} \ \mathrm{d}y = \pi i r \ \int _{0}^{\infty } \frac{e^{-\pi r^{2} u}}{\sqrt{u+t}} \ \mathrm{d}u \end{aligned}$$

for $r \in \mathbb {R}$, $r \ne 0$ and $t \in \mathbb {R}$, $t>0$ (see, e.g., [19, Lemma 1.18]), then a straightforward computation yields

$$\begin{aligned}&\sqrt{6t} \ \left( -\frac{1}{\sqrt{3}} \ J_{1} \left( \frac{3 \pi t}{2} \right) - \frac{1}{\sqrt{3}} \ J \left( \frac{3 \pi t}{2} \right) \right) \\&= \ \frac{2 i^{\frac{3}{2}}}{\sqrt{3} \sqrt{i t}} \ \int _{0}^{i \infty } \left( \frac{3 \ \sum _{n \in \mathbb {Z}} \left( 2n+\frac{1}{2} \right) \ e^{6 \pi i (2n+1/2)^{2} z}}{\sqrt{-i \left( z-\frac{1}{it} \right) }} + \frac{\sum _{n \in \mathbb {Z}} \left( 2n+\frac{1}{2} \right) \ e^{\frac{2}{3} \pi i (2n+1/2)^{2} z}}{\sqrt{-i \left( z-\frac{1}{it} \right) }} \right) \ \mathrm{d}z. \end{aligned}$$

The identity above is valid for all $t \in \mathbb {R}$, $t>0$; thus, the identity theorem for holomorphic functions yields that for all $\tau \in \mathbb {H}$ the first component of $R_{(6)}(\tau )$ is equal to

$$\begin{aligned} \frac{2}{\sqrt{3}} \ i^{\frac{3}{2}} \ \int _{0}^{i \infty } \frac{3 \ \sum _{n \in \mathbb {Z}} \left( 2n+\frac{1}{2} \right) \ e^{6 \pi i (2n+1/2)^{2} z} + \sum _{n \in \mathbb {Z}} \left( 2n+\frac{1}{2} \right) \ e^{\frac{2}{3} \pi i (2n+1/2)^{2} z}}{\sqrt{-i (z \tau - 1)}} \ \mathrm{d}z. \end{aligned}$$

To rewrite the numerator in terms of theta functions we note that

$$\begin{aligned} \sum _{n \equiv 2 \ (3)} \left( 2n+\frac{1}{2} \right) \ e^{\frac{2}{3} \pi i (2n+1/2)^{2} z} = -3 \cdot \sum _{n \in \mathbb {Z}} \left( 2n+\frac{1}{2} \right) \ e^{6 \pi i (2n+1/2)^{2} z}. \end{aligned}$$

By a calculation this implies

$$\begin{aligned} 3 \ \sum _{n \in \mathbb {Z}} \left( 2n+\frac{1}{2} \right) \ e^{6 \pi i (2n+1/2)^{2} z} + \sum _{n \in \mathbb {Z}} \left( 2n+\frac{1}{2} \right) \ e^{\frac{2}{3} \pi i (2n+1/2)^{2} z} \\ \quad = \frac{1}{4} \ (\theta _{12,2}(z)+\theta _{12,10}(z)). \end{aligned}$$

Hence the first component of identity (3.3) follows.

Using the appropriate partial fraction decompositions of the appearing functions, the identities for the other components can be verified analogously. For more details we refer the reader to [11]. $\square$

Now we can define a non-holomorphic function $G_{(6)}$ such that $F_{(6),1}-G_{(6)}$ and $F_{(6),2}-G_{(6)}$ are vector valued harmonic weak Maass forms.

#### Definition 3.4

For $$\tau \in \mathbb {H}$$ let

$$\begin{aligned} G_{(6)}(\tau ) := \frac{i}{\sqrt{24}} \ \int _{-\overline{\tau }}^{i \infty } \frac{g_{(6)}(z)}{\sqrt{-i(z+\tau )}} \ \mathrm{d}z, \end{aligned}$$

with $g_{(6)}$ as defined in Lemma 3.3.

#### Lemma 3.5

The function $G_{(6)}$ has the same modular transformation properties under $\tau \mapsto \tau +1$ and $\tau \mapsto -\nicefrac {1}{\tau }$ as the one of $F_{(6),1}$ and $F_{(6),2}$, stated in Lemma 3.2.

#### Proof

Let

$$\begin{aligned} N_{(6)} := \left( \begin{matrix} \zeta _{12}^{-1} &{} 0 &{} 0 &{} 0 &{} 0 &{} 0 \\ 0 &{} i &{} 0 &{} 0 &{} 0 &{} 0 \\ 0 &{} 0 &{} 0 &{} \zeta _{48}^{-1} &{} 0 &{} 0 \\ 0 &{} 0 &{} \zeta _{48}^{-1} &{} 0 &{} 0 &{} 0 \\ 0 &{} 0 &{} 0 &{} 0 &{} 0 &{} \zeta _{16}^{-3} \\ 0 &{} 0 &{} 0 &{} 0 &{} \zeta _{16}^{-3} &{} 0 \end{matrix}\right) . \end{aligned}$$

We use formula (2.2) with *z* replaced by $z-1$ and obtain

$$\begin{aligned} g_{(6)}(z-1) = N_{(6)} \ g_{(6)}(z). \end{aligned}$$

This leads to the identity

$$\begin{aligned} G_{(6)}(\tau +1) = N_{(6)} \ G_{(6)}(\tau ) \end{aligned}$$

by a transformation of the defining integral.

Using formula (2.3) we get the transformation behaviour

$$\begin{aligned} g_{(6)} \left( -\frac{1}{z} \right) = (-iz)^{\frac{3}{2}} \ (-M_{(6)}) \ g_{(6)}(z). \end{aligned}$$

Via an integral transformation this gives us the identities

$$\begin{aligned} \frac{1}{\sqrt{-i \tau }} \ G_{(6)} \left( -\frac{1}{\tau } \right) = -\frac{i}{\sqrt{24}} \ \int _{0}^{-\overline{\tau }} \frac{M_{(6)} \ g_{(6)}(u)}{\sqrt{-i(u+\tau )}} \ \mathrm{d}u \end{aligned}$$

and

$$\begin{aligned} \frac{1}{\sqrt{-i \tau }} \ G_{(6)} \left( -\frac{1}{\tau } \right) - M_{(6)} \ G_{(6)}(\tau ) = R_{(6)}(\tau ). \end{aligned}$$

$$\square $$

Using the last lemma we now get that $F_{(6),1}$ and $F_{(6),2}$ are the holomorphic parts of two vector valued harmonic weak Maass forms of weight $\nicefrac {1}{2}$.

#### Theorem 3.6

The functions $H_{(6),1}$ and $H_{(6),2}$, defined for $\tau \in \mathbb {H}$ by

$$\begin{aligned} H_{(6),1}(\tau )&:=F_{(6),1}(\tau )-G_{(6)}(\tau ), \\ H_{(6),2}(\tau )&:=F_{(6),2}(\tau )-G_{(6)}(\tau ), \end{aligned}$$

are vector valued harmonic weak Maass forms of weight $\nicefrac {1}{2}$ for the metaplectic group $\text {Mp} _{2}(\mathbb {Z})$.

For $j=1,2$ and $\tau \in \mathbb {H}$ we have

3.4 $$\begin{aligned} H_{(6),j}(\tau +1) = \left( \begin{matrix} \zeta _{12}^{-1} &{} 0 &{} 0 &{} 0 &{} 0 &{} 0 \\ 0 &{} i &{} 0 &{} 0 &{} 0 &{} 0 \\ 0 &{} 0 &{} 0 &{} \zeta _{48}^{-1} &{} 0 &{} 0 \\ 0 &{} 0 &{} \zeta _{48}^{-1} &{} 0 &{} 0 &{} 0 \\ 0 &{} 0 &{} 0 &{} 0 &{} 0 &{} \zeta _{16}^{-3} \\ 0 &{} 0 &{} 0 &{} 0 &{} \zeta _{16}^{-3} &{} 0 \end{matrix}\right) \ H_{(6),j}(\tau ) \end{aligned}$$

and

3.5 $$\begin{aligned} H_{(6),j} \left( -\frac{1}{\tau } \right) = \sqrt{-i \tau } \ \left( \begin{matrix} 0 &{} 0 &{} \frac{1}{\sqrt{3}} &{} 0 &{} \sqrt{\frac{2}{3}} &{} 0 \\ 0 &{} 0 &{} \sqrt{\frac{2}{3}} &{} 0 &{} -\frac{1}{\sqrt{3}} &{} 0 \\ \frac{1}{\sqrt{3}} &{} \sqrt{\frac{2}{3}} &{} 0 &{} 0 &{} 0 &{} 0 \\ 0 &{} 0 &{} 0 &{} \frac{1}{\sqrt{3}} &{} 0 &{} -\sqrt{\frac{2}{3}} \\ \sqrt{\frac{2}{3}} &{} -\frac{1}{\sqrt{3}} &{} 0 &{} 0 &{} 0 &{} 0 \\ 0 &{} 0 &{} 0 &{} -\sqrt{\frac{2}{3}} &{} 0 &{} -\frac{1}{\sqrt{3}} \end{matrix}\right) \ H_{(6),j}(\tau ). \end{aligned}$$

#### Corollary 3.7

We have $$\xi _{1/2}(H_{(6),1})(\tau )=\xi _{1/2}(H_{(6),2})(\tau )=-\frac{1}{\sqrt{12}} \ g_{(6)}(\tau )$$.

Now we know the transformation behaviour of the functions $H_{(6),1}, H_{(6),2}$ under the generators of the modular group as well as the explicit representations to which they transform. We will see now that we can use the transformation properties in Theorem 3.6 to obtain two functions that transform to the Weil representation.

More precisely, we consider the lattice *L* defined at the beginning of Sect. 2.1 with $N=12$, and its associated Weil representation (2.1). We find the following result:

#### Lemma 3.8

Suppose that the function $H=(h_{0},h_{1},h_{2},h_{3},h_{4},h_{5})^{T}$ satisfies the transformation properties (3.4) and (3.5) in Theorem 3.6. Then the function

$$\begin{aligned} \widetilde{H} :=&\sqrt{2} \ h_{0} \ [-(\mathfrak {e}_{2}-\mathfrak {e}_{22})-(\mathfrak {e}_{10}-\mathfrak {e}_{14})] + 2 \ h_{1} \ [-(\mathfrak {e}_{6}-\mathfrak {e}_{18})] \\&+ (h_{2}+h_{3}) \ [(\mathfrak {e}_{1}-\mathfrak {e}_{23})-(\mathfrak {e}_{7}-\mathfrak {e}_{17})] + (h_{2}-h_{3}) \ [(\mathfrak {e}_{5}-\mathfrak {e}_{19})-(\mathfrak {e}_{11}-\mathfrak {e}_{13})] \\&+ \sqrt{2} \ (h_{4}+h_{5}) \ (\mathfrak {e}_{3}-\mathfrak {e}_{21}) + \sqrt{2} \ (h_{4}-h_{5}) \ [-(\mathfrak {e}_{9}-\mathfrak {e}_{15})] \end{aligned}$$

transforms like a vector valued modular form of weight $\nicefrac {1}{2}$ for the dual Weil representation $\overline{\rho }_{L}$ considered above.

From the last lemma we immediately obtain two vector valued harmonic weak Maass forms $\widetilde{H}_{(6),1}$, $\widetilde{H}_{(6),2}$ of weight $\nicefrac {1}{2}$ for $\text {Mp}_{2}(\mathbb {Z})$ and the dual Weil representation $\overline{\rho }_{L}$ of level $N=12$, if we apply the lemma for $H=H_{(6),1}$ and $H=H_{(6),2}$, respectively. Hence $\widetilde{H}_{(6),1}, \widetilde{H}_{(6),2} \in H_{1/2,\overline{\rho }_{L}}^{+}$.

Now we come back to our initial functions $H_{(6),1}$ and $H_{(6),2}$ and want to relate their components to scalar valued harmonic weak Maass forms. In order to do that we consider the congruence subgroup

$$\begin{aligned} \Gamma (6) = \left\{ \left( \begin{matrix} a &{} b \\ c &{} d \\ \end{matrix}\right) \in \text {SL}_{2}(\mathbb {Z}) \ \Big | \ b \equiv c \equiv 0 \ (6), \ a \equiv d \equiv 1 \ (6) \right\} . \end{aligned}$$

With the use of Sage [16] we determined a system of generators for this group, decomposed the generators into products of *S* and *T*, and multiplied the corresponding matrices from Theorem 3.6 according to these products, to obtain the transformation properties of $H_{(6),1}$ and $H_{(6),2}$ under all generators. All of the appearing transformation matrices are diagonal, so we get:

#### Theorem 3.9

For $j=1,2$ the components of the vector valued harmonic weak Maass form $H_{(6),j}$ are scalar valued harmonic weak Maass forms of weight $\nicefrac {1}{2}$ for the subgroup

$$\begin{aligned} \{(\gamma ,\phi ) \in \text {Mp} _{2}(\mathbb {Z}) \ | \ \gamma \in \Gamma (6) \} \end{aligned}$$

of the metaplectic group $\text {Mp} _{2}(\mathbb {Z})$.

Hence the sixth-order mock theta functions $\sigma ,\rho ,\phi ,\psi ,\mu ,\lambda ,\nu$ and $\xi$ are the holomorphic parts of scalar valued harmonic weak Maass forms.

#### Remark 3.10

The $\xi$-images of the harmonic weak Maass forms in Theorem 3.9 can be easily obtained from Corollary 3.7 by looking at the components of $\xi _{1/2}(H_{(6),1})(\tau )$ and $\xi _{1/2}(H_{(6),2})(\tau )$.

As an application of the Millson theta lift, we can now compute the coefficients of the treated mock theta functions in terms of traces of singular moduli by writing them as the Millson theta lift of a suitable weakly holomorphic modular form.

#### Definition 3.11

We define the functions

3.6 $$\begin{aligned} e_{(6),1}(z):=\bigg (\frac{\eta (z)\eta (3z)}{\eta (4z)\eta (12z)}\bigg )^2-16\bigg (\frac{\eta (4z)\eta (12z)}{\eta (z)\eta (3z)}\bigg )^2 \end{aligned}$$

and

3.7 $$\begin{aligned} e_{(6),2}(z):=\bigg (\frac{\eta (z)\eta (3z)}{\eta (4z)\eta (12z)}\bigg )^4-16^2\bigg (\frac{\eta (4z)\eta (12z)}{\eta (z)\eta (3z)}\bigg )^4. \end{aligned}$$

These functions are weakly holomorphic modular forms of weight 0, level 12 whose principal parts start with $q^{-1}$ and $q^{-2}$, respectively.

#### Theorem 3.12

Let $e_{(6),1}(z)\in M_0^!(12)$ be defined as in (3.6).

1. For $n\ge 0$ the coefficients $a_\sigma (n)$ of $\sigma (q)$ are given by
  $$\begin{aligned} a_\sigma (n)&=-\frac{i}{4\sqrt{48n-4}}\big (tr ^+_{e_{(6),1}}(4-48n,2)-tr ^-_{e_{(6),1}}(4-48n,2)\big ). \end{aligned}$$
2. For $n\ge 0$ the coefficients $a_\rho (n)$ of $\rho (q)$ are given by
  $$\begin{aligned} a_\rho (n)&=-\frac{i}{4\sqrt{48(n+1)-36}}\\&\qquad \times \big (tr ^+_{e_{(6),1}}(36-48(n+1),6)-tr ^-_{e_{(6),1}}(36-48(n+1),6)\big ). \end{aligned}$$
3. For $n\ge 0$ the coefficients $a_\phi (n)$ of $\phi (q)$ are given by
  $$\begin{aligned} a_\phi (n)&= {\left\{ \begin{array}{ll} \frac{i}{2\sqrt{48n-1}}\big (tr ^+_{e_{(6),1}}(1-48n,1)-tr ^-_{e_{(6),1}}(1-48n,1)\big ),&{}\text { if }n\text { is even,} \\ \frac{i}{2\sqrt{48n-25}}\big (tr ^+_{e_{(6),1}}(25-48n,5)-tr ^-_{e_{(6),1}}(25-48n,5)\big ), &{}\text { if }n\text { is odd.} \end{array}\right. } \end{aligned}$$
4. For $n\ge 0$ the coefficients $a_\psi (n)$ of $\psi (q)$ are given by
  $$\begin{aligned} a_\psi (n)&={\left\{ \begin{array}{ll} \frac{i}{4\sqrt{48n-9}}\big (tr ^+_{e_{(6),1}}(9-48n,3)-tr ^-_{e_{(6),1}}(9-48n,3)\big ), &{}\text {if }n\text { is even,} \\ \frac{i}{-4\sqrt{48(n+1)-81}}\big (tr ^+_{e_{(6),1}}(81-48(n+1),9)-tr ^-_{e_{(6),1}}(81-48(n+1),9)\big ), &{}\text {if }n\text { is odd.} \end{array}\right. } \end{aligned}$$

#### Proof

As already proven before, the function $\widetilde{H}_{(6),1}$ is a vector valued harmonic weak Maass form of weight $\nicefrac {1}{2}$ for the dual Weil representation. Using the series expansion of $\sigma ,\rho ,\phi$ and $\psi$, one immediately sees that its principal part is given by $2 \ q^{-\frac{1}{48}} \ (\mathfrak {e}_1-\mathfrak {e}_7+\mathfrak {e}_{17}-\mathfrak {e}_{23})$. The function $e_{(6),1}$ is an eigenfunction of all Atkin–Lehner involutions, with eigenvalue $+1$ for the operators $W_1$ and $W_3$ and eigenvalue $-1$ for $W_4$ and $W_{12}$. Thus, the Fourier expansions of $e_{(6),1}$ at the cusps of $\Gamma _0(12)$ only differ by a possible minus sign. Then the Millson theta lift maps the function $e_{(6),1}$ to a harmonic weak Maass form of weight $\nicefrac {1}{2}$ transforming with respect to the dual Weil representation, having the same principal part as $\widetilde{H}_{(6),1}$. In the light of Lemma 2.1, this implies that $\widetilde{H}_{(6),1}-\mathcal {I}^M_{1,1}(e_{(6),1},\tau )$ is a cusp form and thus $\widetilde{H}_{(6),1}=\mathcal {I}^M_{1,1}(e_{(6),1},\tau )$ as $S_{1/2,\overline{\rho }_L}=\{0\}$. Using the result of Theorem 2.2, the holomorphic coefficients of $\mathcal {I}^M_{1,1}(e_{(6),1},\tau )$ at $q^{(48n-r^2)/48}\mathfrak {e}_r$ for $r^2-48n<0$ are given by

$$\begin{aligned} \frac{i}{\sqrt{48n-r^2}}\big (tr ^+_{e_{(6),1}}(r^2-48n,r)-tr ^-_{e_{(6),1}}(r^2-48n,r)\big ). \end{aligned}$$

Comparing the coefficients of the holomorphic parts of both $\widetilde{H}_{(6),1}$ and $\mathcal {I}^M_{1,1}(e_{(6),1},\tau )$ yields the stated formulas. $\square$

#### Theorem 3.13

Let $e_{(6),1}(z)\in M_0^!(12)$ and $e_{(6),2}(z)\in M_0^!(12)$ be defined as in (3.6) and (3.7) and put $E_{(6)}(z):=e_{(6),2}(z)+3e_{(6),1}(z)$.

1. For $n\ge 0$ the coefficients $a_{2\mu }(n)$ of $2\mu (q)$ are given by
  $$\begin{aligned} a_{2\mu }(n)&=\frac{i}{2\sqrt{48n-4}}\big (tr ^+_{E_{(6)}}(4-48n,2)-tr ^-_{E_{(6)}}(4-48n,2)\big ). \end{aligned}$$
2. For $n\ge 0$ the coefficients $a_\lambda (n)$ of $\lambda (q)$ are given by
  $$\begin{aligned} a_\lambda (n)&=\frac{i}{4\sqrt{48n-36}}\big (tr ^+_{E_{(6)}}(36-48n,6)-tr ^-_{E_{(6)}}(36-48n,6)\big ). \end{aligned}$$
3. For $n\ge 0$ the coefficients $a_\nu (n)$ of $\nu (q)$ are given by
  $$\begin{aligned} a_\nu (n)&= {\left\{ \begin{array}{ll} -\frac{i}{8\sqrt{48n-1}}\big (tr ^+_{E_{(6)}}(1-48n,1)-tr ^-_{E_{(6)}}(1-48n,1)\big ),&{}\text { if }n\text { is even,} \\ -\frac{i}{8\sqrt{48n-25}}\big (tr ^+_{E_{(6)}}(25-48n,5)-tr ^-_{E_{(6)}}(25-48n,5)\big ),&{}\text { if }n\text { is odd.} \end{array}\right. } \end{aligned}$$
4. For $n\ge 0$ the coefficients $a_\xi (n)$ of $\xi (q)$ are given by
  $$\begin{aligned} a_\xi (n)&={\left\{ \begin{array}{ll} -\frac{i}{16\sqrt{48n-9}}\big (tr ^+_{E_{(6)}}(9-48n,3)-tr ^-_{E_{(6)}}(9-48n,3)\big ),&{}\text { if }n\text { is even,} \\ \frac{i}{16\sqrt{48(n+1)-81}}\big (tr ^+_{E_{(6)}}(81-48(n+1),9)-tr ^-_{E_{(6)}}(81-48(n+1),9)\big ),&{}\text { if }n\text { is odd.} \end{array}\right. } \end{aligned}$$

#### Proof

The proof is analogous to that of Theorem 3.12. $\square$

#### Remark 3.14

The stated formulas were checked numerically using Sage [16].

### Mock theta functions of order 2

In this subsection we consider the mock theta functions *A*, *B* and $\mu$ of order 2 and prove similar results for their completions to harmonic weak Maass forms as in Sect. 3.1. We omit the proofs here since all results of this subsection can be proven analogously to the results of the previous subsection.

#### Definition 3.15

For $\tau \in \mathbb {H}$ we define the vector valued functions

$$\begin{aligned} F_{(2)}(\tau ) := \left( \begin{matrix} 4 \ q^{-\frac{1}{16}} \ A(q^{\frac{1}{2}}) \\ 4 \ q^{-\frac{1}{16}} \ A(-q^{\frac{1}{2}}) \\ \sqrt{8} \ q^{\frac{1}{4}} \ B(q^{\frac{1}{2}}) \\ \sqrt{8} \ q^{\frac{1}{4}} \ B(-q^{\frac{1}{2}}) \\ q^{-\frac{1}{16}} \ \mu (q^{\frac{1}{2}}) \\ q^{-\frac{1}{16}} \ \mu (-q^{\frac{1}{2}}) \end{matrix}\right) , \end{aligned}$$

where $$q=e^{2 \pi i \tau }$$, and

$$\begin{aligned} G_{(2)}(\tau ) := \frac{i}{\sqrt{2}} \ \int _{-\overline{\tau }}^{i \infty } \frac{g_{(2)}(z)}{\sqrt{-i(z+\tau )}} \ \mathrm{d}z, \end{aligned}$$

where $g_{(2)}$ is the vector $(g_{(2),0},\dots ,g_{(2),5})^{T}$ with components

$$\begin{aligned} g_{(2),0}(z)&:= \theta _{4,1}(z)+\theta _{4,3}(z), \\ g_{(2),1}(z)&:= \theta _{4,1}(z)-\theta _{4,3}(z), \\ g_{(2),2}(z)&:= \sqrt{2} \ \theta _{4,2}(z), \\ g_{(2),3}(z)&:= -\sqrt{2} \ \theta _{4,2}(z), \\ g_{(2),4}(z)&:= -(\theta _{4,1}(z)-\theta _{4,3}(z)), \\ g_{(2),5}(z)&:= -(\theta _{4,1}(z)+\theta _{4,3}(z)). \end{aligned}$$

The so-defined functions $F_{(2)}$ and $G_{(2)}$ have the same modular transformation properties. As before we can consider $F_{(2)}-G_{(2)}$ which will be a vector valued harmonic weak Maass form as the following theorem states:

#### Theorem 3.16

The function $H_{(2)}$, defined for $\tau \in \mathbb {H}$ by

$$\begin{aligned} H_{(2)}(\tau ):=F_{(2)}(\tau )-G_{(2)}(\tau ) \end{aligned}$$

is a vector valued harmonic weak Maass form of weight $\nicefrac {1}{2}$ for the metaplectic group $\text {Mp} _{2}(\mathbb {Z})$.

For $\tau \in \mathbb {H}$ we have

3.8 $$\begin{aligned} H_{(2)}(\tau +1) = \left( \begin{matrix} 0 &{} \zeta _{16}^{-1} &{} 0 &{} 0 &{} 0 &{} 0 \\ \zeta _{16}^{-1} &{} 0 &{} 0 &{} 0 &{} 0 &{} 0 \\ 0 &{} 0 &{} 0 &{} i &{} 0 &{} 0 \\ 0 &{} 0 &{} i &{} 0 &{} 0 &{} 0 \\ 0 &{} 0 &{} 0 &{} 0 &{} 0 &{} \zeta _{16}^{-1} \\ 0 &{} 0 &{} 0 &{} 0 &{} \zeta _{16}^{-1} &{} 0 \end{matrix}\right) \ H_{(2)}(\tau ) \end{aligned}$$

and

3.9 $$\begin{aligned} H_{(2)} \left( -\frac{1}{\tau } \right) = \sqrt{-i \tau } \ \left( \begin{matrix} 0 &{} 0 &{} 0 &{} 0 &{} 0 &{} 1 \\ 0 &{} 0 &{} 0 &{} 1 &{} 0 &{} 0 \\ 0 &{} 0 &{} 0 &{} 0 &{} 1 &{} 0 \\ 0 &{} 1 &{} 0 &{} 0 &{} 0 &{} 0 \\ 0 &{} 0 &{} 1 &{} 0 &{} 0 &{} 0 \\ 1 &{} 0 &{} 0 &{} 0 &{} 0 &{} 0 \end{matrix}\right) \ H_{(2)}(\tau ). \end{aligned}$$

#### Corollary 3.17

We have $$\xi _{1/2}(H_{(2)})(\tau )=-g_{(2)}(\tau )$$.

After we have constructed a vector valued harmonic weak Maass form that contains mock theta functions of order 2, we again take a closer look at its components. We consider

$$\begin{aligned} \Gamma (2) = \left\{ \left( \begin{matrix} a &{} b \\ c &{} d \\ \end{matrix}\right) \in \text {SL}_{2}(\mathbb {Z}) \ \Big | \ b \equiv c \equiv 0 \ (2), \ a \equiv d \equiv 1 \ (2) \right\} , \end{aligned}$$

the principal congruence subgroup of level 2, and obtain the following result:

#### Theorem 3.18

The components of the vector valued harmonic weak Maass form $H_{(2)}$ are scalar valued harmonic weak Maass forms of weight $\nicefrac {1}{2}$ for the subgroup

$$\begin{aligned} \{(\gamma ,\phi ) \in \text {Mp} _{2}(\mathbb {Z}) \ | \ \gamma \in \Gamma (2) \} \end{aligned}$$

of the metaplectic group $\text {Mp} _{2}(\mathbb {Z})$.

So we have interpreted all second-order mock theta functions as the holomorphic part of a scalar valued harmonic weak Maass form.

#### Remark 3.19

As in the previous section, the $\xi$-images of the harmonic weak Maass forms in Theorem 3.18 follow immediately from Corollary 3.17.

### Mock theta functions of order 3

We now turn to the mock theta functions $\phi$, $\psi$ and $\nu$ of order 3. As before, we omit proofs in this subsection.

#### Definition 3.20

For $\tau \in \mathbb {H}$ we define the vector valued functions

$$\begin{aligned} F_{(3)}(\tau ) := \left( \begin{matrix} q^{-\frac{1}{48}} \ \phi (q^{\frac{1}{2}}) \\ q^{-\frac{1}{48}} \ \phi (-q^{\frac{1}{2}}) \\ 2 \ q^{-\frac{1}{48}} \ \psi (q^{\frac{1}{2}}) \\ 2 \ q^{-\frac{1}{48}} \ \psi (-q^{\frac{1}{2}}) \\ \sqrt{2} \ q^{\frac{1}{6}} \ \nu (q^{\frac{1}{2}}) \\ \sqrt{2} \ q^{\frac{1}{6}} \ \nu (-q^{\frac{1}{2}}) \end{matrix}\right) , \end{aligned}$$

where $$q=e^{2 \pi i \tau }$$, and

$$\begin{aligned} G_{(3)}(\tau ) := \frac{i}{\sqrt{24}} \ \int _{-\overline{\tau }}^{i \infty } \frac{g_{(3)}(z)}{\sqrt{-i(z+\tau )}} \ \mathrm{d}z, \end{aligned}$$

where $g_{(3)}$ is the vector $(g_{(3),0},\dots ,g_{(3),5})^{T}$ with components

$$\begin{aligned} g_{(3),0}(z)&:= -(\theta _{12,1}(z)+\theta _{12,5}(z)+\theta _{12,7}(z)+\theta _{12,11}(z)), \\ g_{(3),1}(z)&:= -(\theta _{12,1}(z)-\theta _{12,5}(z)+\theta _{12,7}(z)-\theta _{12,11}(z)), \\ g_{(3),2}(z)&:= \theta _{12,1}(z)+\theta _{12,5}(z)+\theta _{12,7}(z)+\theta _{12,11}(z), \\ g_{(3),3}(z)&:= \theta _{12,1}(z)-\theta _{12,5}(z)+\theta _{12,7}(z)-\theta _{12,11}(z), \\ g_{(3),4}(z)&:= -\sqrt{2} \ (\theta _{12,4}(z)+\theta _{12,8}(z)), \\ g_{(3),5}(z)&:= \sqrt{2} \ (\theta _{12,4}(z)+\theta _{12,8}(z)). \end{aligned}$$

Since these two functions have the same modular transformation properties, we find for the function $F_{(3)}-G_{(3)}$:

#### Theorem 3.21

The function $H_{(3)}$, defined for $\tau \in \mathbb {H}$ by

$$\begin{aligned} H_{(3)}(\tau ):=F_{(3)}(\tau )-G_{(3)}(\tau ), \end{aligned}$$

is a vector valued harmonic weak Maass form of weight $\nicefrac {1}{2}$ for the metaplectic group $\text {Mp} _{2}(\mathbb {Z})$.

For $\tau \in \mathbb {H}$ we have

3.10 $$\begin{aligned} H_{(3)}(\tau +1) = \left( \begin{matrix} 0 &{} \zeta _{48}^{-1} &{} 0 &{} 0 &{} 0 &{} 0 \\ \zeta _{48}^{-1} &{} 0 &{} 0 &{} 0 &{} 0 &{} 0 \\ 0 &{} 0 &{} 0 &{} \zeta _{48}^{-1} &{} 0 &{} 0 \\ 0 &{} 0 &{} \zeta _{48}^{-1} &{} 0 &{} 0 &{} 0 \\ 0 &{} 0 &{} 0 &{} 0 &{} 0 &{} \zeta _{6} \\ 0 &{} 0 &{} 0 &{} 0 &{} \zeta _{6} &{} 0 \end{matrix}\right) \ H_{(3)}(\tau ) \end{aligned}$$

and

3.11 $$\begin{aligned} H_{(3)} \left( -\frac{1}{\tau } \right) = \sqrt{-i \tau } \ \left( \begin{matrix} 0 &{} 0 &{} 1 &{} 0 &{} 0 &{} 0 \\ 0 &{} 0 &{} 0 &{} 0 &{} 0 &{} 1 \\ 1 &{} 0 &{} 0 &{} 0 &{} 0 &{} 0 \\ 0 &{} 0 &{} 0 &{} 0 &{} 1 &{} 0 \\ 0 &{} 0 &{} 0 &{} 1 &{} 0 &{} 0 \\ 0 &{} 1 &{} 0 &{} 0 &{} 0 &{} 0 \end{matrix}\right) \ H_{(3)}(\tau ). \end{aligned}$$

#### Corollary 3.22

We have $$\xi _{1/2}(H_{(3)})(\tau )=-\frac{1}{\sqrt{12}} \ g_{(3)}(\tau )$$.

We now want to complete the mock theta functions $\phi ,\psi$ and $\nu$ to scalar valued harmonic weak Maass forms. We again consider the group $\Gamma (2)$ and obtain:

#### Theorem 3.23

The components of the vector valued harmonic weak Maass form $H_{(3)}$ are scalar valued harmonic weak Maass forms of weight $\nicefrac {1}{2}$ for the subgroup

$$\begin{aligned} \{(\gamma ,\phi ) \in \text {Mp} _{2}(\mathbb {Z}) \ | \ \gamma \in \Gamma (2) \} \end{aligned}$$

of the metaplectic group $\text {Mp} _{2}(\mathbb {Z})$.

Thus we have related the mock theta functions $\phi ,\psi$ and $\nu$ to scalar valued harmonic weak Maass forms.

#### Remark 3.24

Again we get the $\xi$-images of the harmonic weak Maass forms in Theorem 3.23 from Corollary 3.22.

The mock theta functions *f* and $\omega$ of order 3 have already been treated by Zwegers [19], and Bruinier and Schwagenscheidt [7] and we state their results for completeness.

#### Theorem 3.25

[19, Theorem 3.6]. The vector

$$\begin{aligned} F_3(\tau )=\begin{pmatrix} q^{-\frac{1}{24}}f(q)\\ 2\ q^{\frac{1}{3}}\ \omega (q^{\frac{1}{2}})\\ 2 \ q^{\frac{1}{3}}\ \omega (-q^{\frac{1}{2}}) \end{pmatrix} \end{aligned}$$

is the holomorphic part of a harmonic weak Maass form $H_{3}=(h_0,h_1,h_2)^T\in H^+_{1/2}$ of weight $\nicefrac {1}{2}$, transforming as

$$\begin{aligned} H_3(\tau +1)=\begin{pmatrix} \zeta _{24}^{-1}&{}0&{}0\\ 0&{}0&{}\zeta _3\\ 0&{}\zeta _3&{}0 \end{pmatrix} \ H_3(\tau ) \end{aligned}$$

and

$$\begin{aligned} H_3\bigg (-\frac{1}{\tau }\bigg )=\sqrt{-i \tau } \ \begin{pmatrix} 0&{}1&{}0\\ 1&{}0&{}0\\ 0&{}0&{}-1 \end{pmatrix} \ H_3(\tau ). \end{aligned}$$

This result can be used to construct a harmonic weak Maass form that transforms with respect to the dual Weil representation.

#### Lemma 3.26

The function

$$\begin{aligned} \widetilde{H}_3=h_0 \ [\mathfrak {e}_1-\mathfrak {e}_5+\mathfrak {e}_7-\mathfrak {e}_{11}]+(h_2-h_1) \ [\mathfrak {e}_2-\mathfrak {e}_{10}]+(h_1+h_2) \ [-\mathfrak {e}_4+\mathfrak {e}_8] \end{aligned}$$

transforms like a vector valued modular form of weight $\nicefrac {1}{2}$ with respect to the dual Weil representation $\overline{\rho }_L$ of level $N=6$.

Let $E_4$ denote the normalized Eisenstein series of weight 4 for $\text {SL}_2(\mathbb {Z})$. We consider the function

3.12 $$\begin{aligned} e_{(3)}(z):=-\frac{1}{40}\ \frac{E_4(z)+4E_4(2z)-9E_4(3z)-36E_4(6z)}{(\eta (z)\eta (2z)\eta (3z)\eta (6z))^2} \end{aligned}$$

which is a weakly holomorphic modular form of weight 0, level 6 and whose principal part starts with $q^{-1}$.

#### Theorem 3.27

[7, Theorem 3.1]. Let $e_{(3)}\in M_0^!(6)$ be the function defined in (3.12).

1. For $n\ge 1$ the coefficients $a_f(n)$ of *f*(*q*) are given by
  $$\begin{aligned} a_f(q)=\frac{i}{2\sqrt{24n-1}}\big (tr _{e_{(3)}}^+(1-24n,1)-tr _{e_{(3)}}^-(1-24n,1)\big ). \end{aligned}$$
2. For $n\ge 1$ the coefficients $a_\omega (n)$ of $\omega (q)$ are given by
  $$\begin{aligned} a_\omega (q)={\left\{ \begin{array}{ll}\frac{-i}{8\sqrt{24(\frac{n}{2}+1)-16}}\big (tr _{e_{(3)}}^+(16-24(\frac{n}{2}+1),4)-tr _{e_{(3)}}^-(16-24(\frac{n}{2}+1),4)\big ), &{}\text { if } n \text { is even,}\\ \frac{-i}{8\sqrt{24\frac{n+1}{2}-4}}\big (tr _{e_{(3)}}^+(4-24\frac{n+1}{2},2)-tr _{e_{(3)}}^-(4-24\frac{n+1}{2},2)\big ), &{}\text { if } n \text { is odd.} \end{array}\right. } \end{aligned}$$

### Mock theta functions of order 5

For the mock theta functions of order 5 the necessary completions and their transformation properties have already been studied by Zwegers and Andersen [3, 20], respectively. Using their results we derive algebraic formulas for their coefficients. The proofs are analogous to the corresponding proofs in Sect. 3.1.

We define the two matrices

3.13 $$\begin{aligned} N_{(5)}=\begin{pmatrix} \zeta _{60}^{-1}&{}0&{}0&{}0&{}0&{}0\\ 0&{}\zeta _{60}^{11}&{}0&{}0&{}0&{}0\\ 0&{}0&{}0&{}0&{}\zeta _{240}^{-1}&{}0\\ 0&{}0&{}0&{}0&{}0&{}\zeta _{240}^{71}\\ 0&{}0&{}\zeta _{240}^{-1}&{}0&{}0&{}0\\ 0&{}0&{}0&{}\zeta _{240}^{71}&{}0&{}0 \end{pmatrix} \end{aligned}$$

and

3.14 $$\begin{aligned} M_{(5)}=\begin{pmatrix} 0&{}0&{}\sqrt{2} \ \sin (\frac{\pi }{5})&{}\sqrt{2} \ \sin (\frac{2\pi }{5})&{}0&{}0\\ 0&{}0&{}\sqrt{2} \ \sin (\frac{2\pi }{5})&{}-\sqrt{2} \ \sin (\frac{\pi }{5})&{}0&{}0\\ \frac{1}{\sqrt{2}} \ \sin (\frac{\pi }{5})&{}\frac{1}{\sqrt{2}} \ \sin (\frac{2\pi }{5})&{}0&{}0&{}0&{}0\\ \frac{1}{\sqrt{2}} \ \sin (\frac{2\pi }{5})&{}-\frac{1}{\sqrt{2}} \ \sin (\frac{\pi }{5})&{}0&{}0&{}0&{}0\\ 0&{}0&{}0&{}0&{}\sin (\frac{2\pi }{5})&{}\sin (\frac{\pi }{5})\\ 0&{}0&{}0&{}0&{}\sin (\frac{\pi }{5})&{}\sin (\frac{2\pi }{5}) \end{pmatrix}. \end{aligned}$$

#### Theorem 3.28

[20, Proposition 4.10] The vector

$$\begin{aligned} F_{(5),1}(\tau )=\begin{pmatrix} q^{-\frac{1}{60}} \ f_0(q)\\ q^{\frac{11}{60}} \ f_1(q)\\ q^{-\frac{1}{240}}\ \big (-1+F_0(q^{1/2})\big )\\ q^{\frac{71}{240}} \ F_1(q^{1/2})\\ q^{-\frac{1}{240}}\ (-1+F_0\big (-q^{1/2})\big )\\ q^{\frac{71}{240}} \ F_1(-q^{1/2}) \end{pmatrix} \end{aligned}$$

is the holomorphic part of $H_{(5),1}=(f_{4,1},f_{196,1},f_{1,1},f_{169,1},g_{1,1},g_{169,1})^T\in H_{1/2}^+$, which is a harmonic weak Maass form of weight $\nicefrac {1}{2}$, transforming as

3.15 $$\begin{aligned} H_{(5),1}(\tau +1)=N_{(5)} \ H_{(5),1}(\tau ) \end{aligned}$$

and

3.16 $$\begin{aligned} H_{(5),1}\bigg (-\frac{1}{\tau }\bigg )=\sqrt{-i\tau } \ \frac{2}{\sqrt{5}} \ M_{(5)} \ H_{(5),1}(\tau ), \end{aligned}$$

where the matrices $N_{(5)}$ and $M_{(5)}$ are defined as in (3.13) and (3.14).

#### Theorem 3.29

[20, Proposition 4.13]. The vector

$$\begin{aligned} F_{(5),2}(\tau )=\begin{pmatrix} 2 \ q^{-\frac{1}{60}} \ \psi _0(q)\\ 2 \ q^{\frac{11}{60}} \ \psi _1(q)\\ q^{-\frac{1}{240}} \ \varphi _0(-q^{\frac{1}{2}})\\ -q^{-\frac{49}{240}} \ \varphi _1(-q^{\frac{1}{2}})\\ q^{-\frac{1}{240}} \ \varphi _0(q^{\frac{1}{2}})\\ q^{-\frac{49}{240}} \ \varphi _1(q^{\frac{1}{2}}) \end{pmatrix} \end{aligned}$$

is the holomorphic part of $H_{(5),2}=(f_{4,2},f_{196,2},f_{1,2},f_{169,2},g_{1,2},g_{169,2})^T\in H_{1/2}^+$, which is a harmonic weak Maass form of weight $\nicefrac {1}{2}$, transforming as

3.17 $$\begin{aligned} H_{(5),2}(\tau +1)=N_{(5)} \ H_{(5),2}(\tau ) \end{aligned}$$

and

3.18 $$\begin{aligned} H_{(5),2}\bigg (-\frac{1}{\tau }\bigg )=\sqrt{-i\tau } \ \frac{2}{\sqrt{5}} \ M_{(5)} \ H_{(5),2}(\tau ), \end{aligned}$$

where the matrices $N_{(5)}$ and $M_{(5)}$ are defined as in (3.13) and (3.14).

#### Lemma 3.30

[3, Lemma 5] Suppose that $(f_{4,1},f_{196,1},f_{1,1},f_{169,1},g_{1,1},g_{169,1})^T$ transforms with the representation given in Theorem 3.28, and that $(f_{4,2},f_{196,2},f_{1,2},f_{169,2},g_{1,2},g_{169,2})^T$ transforms with the representation given in Theorem 3.29. For $j=1,2$ we define the function

$$\begin{aligned} \widetilde{H}_{(5),j}&=\sum \limits _{\begin{array}{c} 0<r<60\\ r\equiv \pm 1\ (10)\\ \gcd (r,60)=1 \end{array}}(a_r \ f_{1,j}+b_r \ g_{1,j}) \ (\mathfrak {e}_r-\mathfrak {e}_{-r})-\sum \limits _{\begin{array}{c} 0<r<60\\ r\equiv \pm 2\ (10)\\ \gcd (r,60)=2 \end{array}}f_{4,j} \ (\mathfrak {e}_r-\mathfrak {e}_{-r})\\ {}&+\sum \limits _{\begin{array}{c} 0<r<60\\ r\equiv \pm 3\ (10)\\ \gcd (r,60)=1 \end{array}}(a_r \ f_{169,j}+b_r \ g_{169,j}) \ (\mathfrak {e}_r-\mathfrak {e}_{-r})\\&-\sum \limits _{\begin{array}{c} 0<r<60\\ r\equiv \pm 4\ (10)\\ \gcd (r,60)=2 \end{array}}f_{196,j} \ (\mathfrak {e}_r-\mathfrak {e}_{-r}), \end{aligned}$$

where

$$\begin{aligned} a_r={\left\{ \begin{array}{ll}+1\quad \text {if }0<r<30,\\ -1\quad \text {otherwise,} \end{array}\right. }\quad \text {and}\quad b_r={\left\{ \begin{array}{ll}+1\quad \text {if }r\equiv \pm 1,\pm 13\ (60),\\ -1\quad \text {otherwise}. \end{array}\right. } \end{aligned}$$

Then $\widetilde{H}_{(5),1}\in H_{1/2,\overline{\rho }_L}^+$ and $\widetilde{H}_{(5),2}\in H_{1/2,\overline{\rho }_L}^+$ both transform like a vector valued modular form of weight $\nicefrac {1}{2}$ for the dual Weil representation $\overline{\rho }_L$ of level $N=60$.

#### Definition 3.31

We define the functions

3.19 $$\begin{aligned} e_{(5),1}(z):=\frac{\eta (z)\eta (12z)\eta (15z)\eta (20z)}{\eta (3z)\eta (4z)\eta (5z)\eta (60z)}-\frac{\eta (3z)\eta (4z)\eta (5z)\eta (60z)}{\eta (z)\eta (12z)\eta (15z)\eta (20z)} \end{aligned}$$

and

3.20 $$\begin{aligned} e_{(5),2}:=\bigg ( \frac{\eta (z)\eta (12z)\eta (15z)\eta (20z)}{\eta (3z)\eta (4z)\eta (5z)\eta (60z)}\bigg )^2-\bigg (\frac{\eta (3z)\eta (4z)\eta (5z)\eta (60z)}{\eta (z)\eta (12z)\eta (15z)\eta (20z)}\bigg )^2. \end{aligned}$$

These functions are weakly holomorphic modular forms of weight 0, level 60 whose principal parts start with $q^{-1}$ and $q^{-2}$, respectively.

#### Theorem 3.32

Let $e_{(5),1}(z),e_{(5),2}(z)\in M_0^{!}(60)$ be defined as in (3.19) and (3.20) and put $E_{(5)}(z):=-e_{(5),2}-e_{(5),1}$.

1. For $n\ge 1$ the coefficients $a_{f_0}(n)$ of $f_0(q)$ are given by
  $$\begin{aligned} a_{f_0}(n)=\frac{-i}{2\sqrt{240n-4}}\big (tr _{E_{(5)}}^+(4-240n,2)-tr _{E_{(5)}}^-(4-240n,2)\big ). \end{aligned}$$
2. For $n\ge 1$ the coefficients $a_{f_1}(n)$ of $f_1(q)$ are given by
  $$\begin{aligned} a_{f_1}(n)&=\frac{-i}{2\sqrt{240(n+1)-196}}\big (tr _{E_{(5)}}^+(196-240(n+1),14)\\&\quad -tr _{E_{(5)}}^-(196-240(n+1),14)\big ). \end{aligned}$$
3. For $n\ge 1$ the coefficients $a_{F_0}(n)$ of $F_0(q)$ are given by
  $$\begin{aligned} a_{F_0}(n)={\left\{ \begin{array}{ll} \frac{i}{4\sqrt{240\frac{n}{2}-1}}\big (tr _{E_{(5)}}^+(1-240\frac{n}{2},1)-tr _{E_{(5)}}^-(1-240\frac{n}{2},1)\big ), &{}\text {if } n\ \text {is even}, \\ \frac{i}{4\sqrt{240\frac{n+1}{2}-121}}\big (tr _{E_{(5)}}^+(121-240\frac{n+1}{2},11)-tr _{E_{(5)}}^-(121-240\frac{n+1}{2},11)\big ), &{}\text {if } n\ \text {is odd.} \end{array}\right. } \end{aligned}$$
4. For $n\ge 1$ the coefficients $a_{F_1}(n)$ of $F_1(q)$ are given by
  $$\begin{aligned} a_{F_1}(n)={\left\{ \begin{array}{ll} \frac{i}{4\sqrt{240\frac{n+2}{2}-169}}\big (tr _{E_{(5)}}^+(169-240\frac{n+2}{2},13)-tr _{E_{(5)}}^-(169-240\frac{n+2}{2}),13)\big ),&{}\text {if }n\ \text {is even},\\ \frac{i}{4\sqrt{240\frac{n+1}{2}-49}}\big (tr _{E_{(5)}}^+(49-240\frac{n+1}{2},7)-tr _{E_{(5)}}^-(49-240\frac{n+1}{2},7)\big ),&{}\text {if }n\ \text {is odd.} \end{array}\right. } \end{aligned}$$

#### Theorem 3.33

Let $e_{(5),1}\in M_0^{!}(60)$ be defined as in (3.19).

1. For $n\ge 1$ the coefficients $a_{\psi _0}(n)$ of $\psi _0(q)$ are given by
  $$\begin{aligned} a_{\psi _0}(n)=\frac{-i}{2\sqrt{240n-4}}\big (tr _{e_{(5),1}}^+(4-240n,2)-tr _{e_{(5),1}}^-(4-240n,2)\big ). \end{aligned}$$
2. For $n\ge 1$ the coefficients $a_{\psi _1}(n)$ of $\psi _1(q)$ are given by
  $$\begin{aligned} a_{\psi _1}(n)&=\frac{-i}{2\sqrt{240(n+1)-196}}\big (tr _{e_{(5),1}}^+(196-240(n+1),14)\\&\quad -tr _{e_{(5),1}}^-(196-240(n+1),14)\big ). \end{aligned}$$
3. For $n\ge 1$ the coefficients $a_{\varphi _0}(n)$ of $\varphi _0(q)$ are given by
  $$\begin{aligned} a_{\varphi _0}(n)={\left\{ \begin{array}{ll}\frac{i}{2\sqrt{240\frac{n}{2}-1}}\big (tr _{e_{(5),1}}^+(1-240\frac{n}{2},1)-tr _{e_{(5),1}}^-(1-240\frac{n}{2},1)\big ), &{}\text {if } n\ \text {is even}, \\ \frac{-i}{2\sqrt{240\frac{n+1}{2}-121}}\big (tr _{e_{(5),1}}^+(121-240\frac{n+1}{2},11)-tr _{e_{(5),1}}^-(121-240\frac{n+1}{2},11)\big ), &{}\text {if } n\ \text {is odd.} \end{array}\right. } \end{aligned}$$
4. For $n\ge 1$ the coefficients $a_{\varphi _1}(n)$ of $\varphi _1(q)$ are given by
  $$\begin{aligned} a_{\varphi _1}(n)={\left\{ \begin{array}{ll}\frac{-i}{2\sqrt{240\frac{n}{2}-49}}\big (tr _{e_{(5),1}}^+(49-240\frac{n}{2},7)-tr _{e_{(5),1}}^-(49-240\frac{n}{2},7)\big ), &{}\text {if }n\ \text {is even},\\ \frac{i}{2\sqrt{240\frac{n+1}{2}-169}}\big (tr _{e_{(5),1}}^+(169-240\frac{n+1}{2},13)-tr _{e_{(5),1}}^-(169-240\frac{n+1}{2},13)\big ), &{}\text {if }n\ \text {is odd.} \end{array}\right. } \end{aligned}$$

### Mock theta functions of order 7

Similar to the previous subsection the necessary completion and its transformation behaviour have already been studied by Zwegers and Andersen [4, 20], respectively. We use their results to derive algebraic formulas for the coefficients of the seventh-order mock theta functions.

#### Theorem 3.34

[20, Proposition 4.5]. The vector

$$\begin{aligned} F_{(7)}(\tau )=\begin{pmatrix} q^{-\frac{1}{168}} \ \mathcal {F}_0(q)\\ q^{\frac{47}{168}} \ \mathcal {F}_2(q)\\ q^{-\frac{25}{168}} \ \mathcal {F}_1(q) \end{pmatrix} \end{aligned}$$

is the holomorphic part of a harmonic weak Maass form $H_{(7)}=(f_1,f_{121},f_{25})^T\in H_{1/2}^+$ of weight $\nicefrac {1}{2}$, transforming as

3.21 $$\begin{aligned} H_{(7)}(\tau +1)=\begin{pmatrix} \zeta _{168}^{-1}&{}0&{}0\\ 0&{}\zeta _{168}^{47}&{}0\\ 0&{}0&{}\zeta _{168}^{-25} \end{pmatrix} \ H_{(7)}(\tau ) \end{aligned}$$

and

3.22 $$\begin{aligned} H_{(7)}\bigg (-\frac{1}{\tau }\bigg )=\sqrt{-i\tau } \ \frac{2}{\sqrt{7}} \ \begin{pmatrix} \sin (\frac{\pi }{7})&{}\sin (\frac{3\pi }{7})&{}\sin (\frac{2\pi }{7})\\ \sin (\frac{3\pi }{7})&{}-\sin (\frac{2\pi }{7})&{}\sin (\frac{\pi }{7})\\ \sin (\frac{2\pi }{7})&{}\sin (\frac{\pi }{7})&{}-\sin (\frac{3\pi }{7}) \end{pmatrix} \ H_{(7)}(\tau ). \end{aligned}$$

#### Lemma 3.35

[4, Lemma 4]. Suppose that $(f_1,f_{121},f_{25})^T$ transforms with the representation given in Theorem 3.34. Then the function

$$\begin{aligned} \widetilde{H}_{(7)}=&\sum \limits _{r\in \mathbb {Z}/168\mathbb {Z}}\widetilde{H}_r\mathfrak {e}_r= f_1 \ (\mathfrak {e}_1-\mathfrak {e}_{-1})+f_1 \ (\mathfrak {e}_{41}-\mathfrak {e}_{-41})\\&-\sum \limits _{\begin{array}{c} 2\le r\le 40\\ r^2 \ (168)\in \{1,25,121\} \end{array}}f_{r^2} \ (\mathfrak {e}_r-\mathfrak {e}_{-r}) \end{aligned}$$

transforms like a vector valued modular form of weight $\nicefrac {1}{2}$ for the dual Weil representation $\overline{\rho }_L$ of level $N=42$, so that $\widetilde{H}_{(7)}\in H_{1/2,\overline{\rho }_L}^+$.

#### Definition 3.36

We define the function

3.23 $$\begin{aligned} e_{(7)}(z):=\Bigg (\frac{\eta (z)\eta (6z)\eta (14z)\eta (21z)}{\eta (2z)\eta (3z)\eta (7z)\eta (42z)}\Bigg )^{2}-\Bigg (\frac{\eta (2z)\eta (3z)\eta (7z)\eta (42z)}{\eta (z)\eta (6z)\eta (14z)\eta (21z)}\Bigg )^{2}. \end{aligned}$$

This function is a weakly holomorphic modular form of level 42, weight 0 whose principal part starts with $q^{-1}$.

#### Theorem 3.37

Let $e_{(7)}\in M_0^{!}(42)$ be defined as in (3.23).

1. For $n\ge 1$ the coefficients $a_{\mathcal {F}_0}(n)$ of $\mathcal {F}_0(q)$ are given by
  $$\begin{aligned} a_{\mathcal {F}_0}(n)=\frac{i}{2\sqrt{168n-1}}\big (tr ^+_{e_{(7)}}(1-168n,1)-tr ^-_{e_{(7)}}(1-168n,1)\big ). \end{aligned}$$
2. For $n\ge 1$ the coefficients $a_{\mathcal {F}_1}(n)$ of $\mathcal {F}_1(q)$ are given by
  $$\begin{aligned} a_{\mathcal {F}_1}(n)=\frac{-i}{2\sqrt{168n-25}}\big (tr ^+_{e_{(7)}}(25-168n,5)-tr ^-_{e_{(7)}}(25-168n,5)\big ). \end{aligned}$$
3. For $n\ge 1$ the coefficients $a_{\mathcal {F}_2}(n)$ of $\mathcal {F}_2(q)$ are given by
  $$\begin{aligned} a_{\mathcal {F}_2}(n)&=\frac{-i}{2\sqrt{168(n+1)-121}}\big (tr ^+_{e_{(7)}}(121-168(n+1),11)\\&\quad -tr ^-_{e_{(7)}}(121-168(n+1),11)\big ). \end{aligned}$$

### Mock theta functions of order 8

We now turn to the mock theta functions $S_{0}$, $S_{1}$, $T_{0}$, $T_{1}$, $U_{0}$, $U_{1}$, $V_{0}$ and $V_{1}$ of order 8. They have the following linear relations between them which are an easy consequence of the identities that are, e.g., given as (1.7) and (1.8) in [9].

#### Lemma 3.38

We have

$$\begin{aligned} q^{-\frac{1}{32}} \ U_{0}(q^{\frac{1}{4}})&= q^{-\frac{1}{32}} \ S_{0}(q^{\frac{1}{2}}) + q^{\frac{7}{32}} \ S_{1}(q^{\frac{1}{2}}), \\ q^{-\frac{1}{32}} \ U_{0}(-q^{\frac{1}{4}})&= q^{-\frac{1}{32}} \ S_{0}(q^{\frac{1}{2}}) - q^{\frac{7}{32}} \ S_{1}(q^{\frac{1}{2}}), \\ q^{-\frac{1}{32}} \ U_{1}(q^{\frac{1}{4}})&= q^{-\frac{1}{32}} \ T_{0}(q^{\frac{1}{2}}) + q^{\frac{7}{32}} \ T_{1}(q^{\frac{1}{2}}), \\ q^{-\frac{1}{32}} \ U_{1}(-q^{\frac{1}{4}})&= q^{-\frac{1}{32}} \ T_{0}(q^{\frac{1}{2}}) - q^{\frac{7}{32}} \ T_{1}(q^{\frac{1}{2}}). \end{aligned}$$

#### Definition 3.39

For $\tau \in \mathbb {H}$ we define the vector valued functions

$$\begin{aligned} F_{(8)}(\tau ) := \left( \begin{matrix} V_{0}(q^{\frac{1}{2}}) \\ V_{0}(-q^{\frac{1}{2}}) \\ \sqrt{8} \ q^{-\frac{1}{8}} \ V_{1}(q^{\frac{1}{2}}) \\ \sqrt{8} \ q^{-\frac{1}{8}} \ V_{1}(-q^{\frac{1}{2}}) \\ \sqrt{2} \ q^{-\frac{1}{32}} \ S_{0}(q^{\frac{1}{2}}) \\ \sqrt{2} \ q^{-\frac{1}{32}} \ S_{0}(-q^{\frac{1}{2}}) \\ \sqrt{2} \ q^{\frac{7}{32}} \ S_{1}(q^{\frac{1}{2}}) \\ \sqrt{2} \ q^{\frac{7}{32}} \ S_{1}(-q^{\frac{1}{2}}) \\ \sqrt{8} \ q^{-\frac{1}{32}} \ T_{0}(q^{\frac{1}{2}}) \\ \sqrt{8} \ q^{-\frac{1}{32}} \ T_{0}(-q^{\frac{1}{2}}) \\ \sqrt{8} \ q^{\frac{7}{32}} \ T_{1}(q^{\frac{1}{2}}) \\ \sqrt{8} \ q^{\frac{7}{32}} \ T_{1}(-q^{\frac{1}{2}}) \end{matrix}\right) , \end{aligned}$$

where $$q=e^{2 \pi i \tau }$$, and

$$\begin{aligned} G_{(8)}(\tau ) := \frac{i}{\sqrt{8}} \ \int _{-\overline{\tau }}^{i \infty } \frac{g_{(8)}(z)}{\sqrt{-i(z+\tau )}} \ \mathrm{d}z, \end{aligned}$$

where $g_{(8)}$ is the vector $(g_{(8),0},\dots ,g_{(8),11})^{T}$ with components

$$\begin{aligned} g_{(8),0}(z)&:= \sqrt{2} \ \theta _{8,4}(z), \\ g_{(8),1}(z)&:= -\sqrt{2} \ \theta _{8,4}(z), \\ g_{(8),2}(z)&:= \theta _{8,2}(z)+\theta _{8,6}(z), \\ g_{(8),3}(z)&:= \theta _{8,2}(z)+\theta _{8,6}(z), \\ g_{(8),4}(z)&:= -(\theta _{8,1}(z)-\theta _{8,7}(z)), \\ g_{(8),5}(z)&:= -(\theta _{8,1}(z)+\theta _{8,7}(z)), \\ g_{(8),6}(z)&:= \theta _{8,3}(z)-\theta _{8,5}(z), \\ g_{(8),7}(z)&:= -(\theta _{8,3}(z)+\theta _{8,5}(z)), \\ g_{(8),8}(z)&:= \theta _{8,1}(z)-\theta _{8,7}(z), \\ g_{(8),9}(z)&:= \theta _{8,1}(z)+\theta _{8,7}(z), \\ g_{(8),10}(z)&:= -(\theta _{8,3}(z)-\theta _{8,5}(z)), \\ g_{(8),11}(z)&:= \theta _{8,3}(z)+\theta _{8,5}(z). \end{aligned}$$

Again the so-defined functions have the same modular transformation properties. Considering the function $F_{(8)}-G_{(8)}$ leads to the following theorem:

#### Theorem 3.40

The function $H_{(8)}$, defined for $\tau \in \mathbb {H}$ by

$$\begin{aligned} H_{(8)}(\tau ):=F_{(8)}(\tau )-G_{(8)}(\tau ) \ \ \ \ \ (\tau \in \mathbb {H}), \end{aligned}$$

is a vector valued harmonic Maass form of weight $\nicefrac {1}{2}$ for the metaplectic group $\text {Mp} _{2}(\mathbb {Z})$.

For $\tau \in \mathbb {H}$ we have

3.24 $$\begin{aligned} H_{(8)}(\tau +1) = N_{(8)} \ H_{(8)}(\tau ) \end{aligned}$$

and

3.25 $$\begin{aligned} H_{(8)} \left( -\frac{1}{\tau } \right) = \sqrt{-i \tau } \ M_{(8)} \ H_{(8)}(\tau ), \end{aligned}$$

where the transformation matrices $N_{(8)}$ and $M_{(8)}$ are defined as

$$\begin{aligned} N_{(8)} := \left( \begin{array}{cccccccccccc} 0 &{} 1 &{} 0 &{} 0 &{} 0 &{} 0 &{} 0 &{} 0 &{} 0 &{} 0 &{} 0 &{} 0 \\ 1 &{} 0 &{} 0 &{} 0 &{} 0 &{} 0 &{} 0 &{} 0 &{} 0 &{} 0 &{} 0 &{} 0 \\ 0 &{} 0 &{} 0 &{} \zeta _{8}^{-1} &{} 0 &{} 0 &{} 0 &{} 0 &{} 0 &{} 0 &{} 0 &{} 0 \\ 0 &{} 0 &{} \zeta _{8}^{-1} &{} 0 &{} 0 &{} 0 &{} 0 &{} 0 &{} 0 &{} 0 &{} 0 &{} 0 \\ 0 &{} 0 &{} 0 &{} 0 &{} 0 &{} \zeta _{32}^{-1} &{} 0 &{} 0 &{} 0 &{} 0 &{} 0 &{} 0 \\ 0 &{} 0 &{} 0 &{} 0 &{} \zeta _{32}^{-1} &{} 0 &{} 0 &{} 0 &{} 0 &{} 0 &{} 0 &{} 0 \\ 0 &{} 0 &{} 0 &{} 0 &{} 0 &{} 0 &{} 0 &{} \zeta _{32}^{7} &{} 0 &{} 0 &{} 0 &{} 0 \\ 0 &{} 0 &{} 0 &{} 0 &{} 0 &{} 0 &{} \zeta _{32}^{7} &{} 0 &{} 0 &{} 0 &{} 0 &{} 0 \\ 0 &{} 0 &{} 0 &{} 0 &{} 0 &{} 0 &{} 0 &{} 0 &{} 0 &{} \zeta _{32}^{-1} &{} 0 &{} 0 \\ 0 &{} 0 &{} 0 &{} 0 &{} 0 &{} 0 &{} 0 &{} 0 &{} \zeta _{32}^{-1} &{} 0 &{} 0 &{} 0 \\ 0 &{} 0 &{} 0 &{} 0 &{} 0 &{} 0 &{} 0 &{} 0 &{} 0 &{} 0 &{} 0 &{} \zeta _{32}^{7} \\ 0 &{} 0 &{} 0 &{} 0 &{} 0 &{} 0 &{} 0 &{} 0 &{} 0 &{} 0 &{} \zeta _{32}^{7} &{} 0 \end{array}\right) \end{aligned}$$

and

$$\begin{aligned} M_{(8)} := \left( \begin{array}{cccccccccccc} 0 &{} 0 &{} 0 &{} 0 &{} \frac{1}{\sqrt{2}} &{} 0 &{} \frac{1}{\sqrt{2}} &{} 0 &{} 0 &{} 0 &{} 0 &{} 0 \\ 0 &{} 0 &{} 0 &{} 0 &{} 0 &{} 0 &{} 0 &{} 0 &{} \frac{1}{\sqrt{2}} &{} 0 &{} \frac{1}{\sqrt{2}} &{} 0 \\ 0 &{} 0 &{} 0 &{} 0 &{} \frac{1}{\sqrt{2}} &{} 0 &{} -\frac{1}{\sqrt{2}} &{} 0 &{} 0 &{} 0 &{} 0 &{} 0 \\ 0 &{} 0 &{} 0 &{} 0 &{} 0 &{} 0 &{} 0 &{} 0 &{} -\frac{1}{\sqrt{2}} &{} 0 &{} \frac{1}{\sqrt{2}} &{} 0 \\ \frac{1}{\sqrt{2}} &{} 0 &{} \frac{1}{\sqrt{2}} &{} 0 &{} 0 &{} 0 &{} 0 &{} 0 &{} 0 &{} 0 &{} 0 &{} 0 \\ 0 &{} 0 &{} 0 &{} 0 &{} 0 &{} 0 &{} 0 &{} 0 &{} 0 &{} \frac{\sqrt{2-\sqrt{2}}}{2} &{} 0 &{} \frac{\sqrt{2+\sqrt{2}}}{2} \\ \frac{1}{\sqrt{2}} &{} 0 &{} -\frac{1}{\sqrt{2}} &{} 0 &{} 0 &{} 0 &{} 0 &{} 0 &{} 0 &{} 0 &{} 0 &{} 0 \\ 0 &{} 0 &{} 0 &{} 0 &{} 0 &{} 0 &{} 0 &{} 0 &{} 0 &{} \frac{\sqrt{2+\sqrt{2}}}{2} &{} 0 &{} -\frac{\sqrt{2-\sqrt{2}}}{2} \\ 0 &{} \frac{1}{\sqrt{2}} &{} 0 &{} -\frac{1}{\sqrt{2}} &{} 0 &{} 0 &{} 0 &{} 0 &{} 0 &{} 0 &{} 0 &{} 0 \\ 0 &{} 0 &{} 0 &{} 0 &{} 0 &{} \frac{\sqrt{2-\sqrt{2}}}{2} &{} 0 &{} \frac{\sqrt{2+\sqrt{2}}}{2} &{} 0 &{} 0 &{} 0 &{} 0 \\ 0 &{} \frac{1}{\sqrt{2}} &{} 0 &{} \frac{1}{\sqrt{2}} &{} 0 &{} 0 &{} 0 &{} 0 &{} 0 &{} 0 &{} 0 &{} 0 \\ 0 &{} 0 &{} 0 &{} 0 &{} 0 &{} \frac{\sqrt{2+\sqrt{2}}}{2} &{} 0 &{} -\frac{\sqrt{2-\sqrt{2}}}{2} &{} 0 &{} 0 &{} 0 &{} 0 \end{array}\right) . \end{aligned}$$

#### Corollary 3.41

We have $$\xi _{1/2}(H_{(8)})(\tau )=-\frac{1}{2} \ g_{(8)}(\tau )$$.

In the following we consider the congruence subgroup

$$\begin{aligned} \Gamma (8) = \left\{ \left( \begin{matrix} a &{} b \\ c &{} d \\ \end{matrix}\right) \in \text {SL}_{2}(\mathbb {Z}) \ \Big | \ b \equiv c \equiv 0 \ (8), \ a \equiv d \equiv 1 \ (8) \right\} . \end{aligned}$$

This leads to:

#### Theorem 3.42

The components of the vector valued harmonic weak Maass form $H_{(8)}$ are scalar valued harmonic weak Maass forms of weight $\nicefrac {1}{2}$ for the subgroup

$$\begin{aligned} \{(\gamma ,\phi ) \in \text {Mp} _{2}(\mathbb {Z}) \ | \ \gamma \in \Gamma (8) \} \end{aligned}$$

of the metaplectic group $\text {Mp} _{2}(\mathbb {Z})$.

#### Remark 3.43

As before, the $\xi$-images of the harmonic weak Maass forms in Theorem 3.42 can be directly obtained from Corollary 3.41.

Finally we consider the yet omitted mock theta functions $U_{0}$ and $U_{1}$. Using their relations to $S_{0},S_{1},T_{0}$ and $T_{1}$ in Lemma 3.38 and denoting the components of $H_{(8)}$ by $h_{(8),0},\dots ,h_{(8),11}$ gives us

$$\begin{aligned} h_{(8),4}(\tau ) \pm h_{(8),6}(\tau )&= q^{-\frac{1}{32}} \ U_{0}( \pm q^{\frac{1}{4}}) \\&+ \frac{i}{4} \ \int _{-\overline{\tau }}^{i \infty } \frac{\theta _{8,1}(z) \mp \theta _{8,3}(z) \pm \theta _{8,5}(z)-\theta _{8,7}(z)}{\sqrt{-i(z+\tau )}} \ \mathrm{d}z, \\ h_{(8),8}(\tau ) \pm h_{(8),10}(\tau )&= q^{-\frac{1}{32}} \ U_{1}(\pm q^{\frac{1}{4}}) \\&+ \frac{i}{8} \ \int _{-\overline{\tau }}^{i \infty } \frac{-\theta _{8,1}(z) \pm \theta _{8,3}(z) \mp \theta _{8,5}(z)+\theta _{8,7}(z)}{\sqrt{-i(z+\tau )}} \ \mathrm{d}z. \end{aligned}$$

It can be shown via Sage [16] that $h_{(8),4}$ and $h_{(8),6}$ have the same transformation behaviour under all generators of $\Gamma (8)$, and also the two functions $h_{(8),8}$ and $h_{(8),10}$ have the same transformation properties under all generators of $\Gamma (8)$. From this and Theorem 3.42 we can conclude:

#### Theorem 3.44

The functions $h_{(8),4} \pm h_{(8),6}$ and $h_{(8),8} \pm h_{(8),10}$ are scalar valued harmonic weak Maass forms of weight $\nicefrac {1}{2}$ for the subgroup

$$\begin{aligned} \{(\gamma ,\phi ) \in \text {Mp} _{2}(\mathbb {Z}) \ | \ \gamma \in \Gamma (8) \} \end{aligned}$$

of the metaplectic group $\text {Mp} _{2}(\mathbb {Z})$.

With the treatment of $U_{0}$ and $U_{1}$ we have now related all eighth-order mock theta functions to scalar valued harmonic weak Maass forms.

#### Remark 3.45

We get the $\xi$-images of the harmonic weak Maass forms in Theorem 3.44 from Corollary 3.41 by adding and subtracting the respective components of $\xi _{1/2}(H_{(8)})(\tau )$.

### Mock theta functions of order 10

The necessary completion and its transformation behaviour has already been studied by Moore [14]. We consider the matrices

3.26 $$\begin{aligned} N_{(10)}:=\begin{pmatrix} 0&{}0&{}\zeta _{10}&{}0&{}0&{}0\\ 0&{}0&{}0&{}\zeta _{10}^{-1}&{}0&{}0\\ \zeta _{10}&{}0&{}0&{}0&{}0&{}0\\ 0&{}\zeta _{10}^{-1}&{}0&{}0&{}0&{}0\\ 0&{}0&{}0&{}0&{}\zeta _{40}^{-1}&{}0\\ 0&{}0&{}0&{}0&{}0&{}\zeta _{40}^{-9} \end{pmatrix} \end{aligned}$$

and

3.27 $$\begin{aligned} M_{(10)}:=\begin{pmatrix} 0&{}0&{}0&{}0&{}\sin (\frac{2\pi }{5})&{}-\sin (\frac{\pi }{5})\\ 0&{}0&{}0&{}0&{}\sin (\frac{\pi }{5})&{}\sin (\frac{2\pi }{5})\\ 0&{}0&{}\sin (\frac{2\pi }{5})&{}\sin (\frac{\pi }{5})&{}0&{}0\\ 0&{}0&{}\sin (\frac{\pi }{5})&{}-\sin (\frac{2\pi }{5})&{}0&{}0\\ \sin (\frac{2\pi }{5})&{}\sin (\frac{\pi }{5})&{}0&{}0&{}0&{}0\\ sin(\frac{\pi }{5})&{}\sin (\frac{2\pi }{5})&{}0&{}0&{}0&{}0 \end{pmatrix}. \end{aligned}$$

#### Theorem 3.46

[14, Theorem 1] The vector

$$\begin{aligned} F_{(10)}(\tau )=\left( \begin{matrix}q^{\frac{1}{10}}\ \phi (q^{\frac{1}{2}})\\ q^{-\frac{1}{10}}\ \psi (q^{\frac{1}{2}})\\ q^{\frac{1}{10}}\ \phi (-q^{\frac{1}{2}})\\ q^{-\frac{1}{10}}\ \psi (-q^{\frac{1}{2}})\\ q^{-\frac{1}{40}}\ X(q)\\ q^{-\frac{9}{40}}\ \chi (q) \end{matrix}\right) \end{aligned}$$

is the holomorphic part of $H_{(10)}=(h_{(10),0},h_{(10),1},h_{(10),2},h_{(10),3},h_{(10),4},h_{(10),5})^T\in H_{1/2}^+$, which is a harmonic weak Maass form of weight $\nicefrac {1}{2}$, transforming as

$$\begin{aligned} H_{(10)}(\tau +1)=N_{(10)} \ H_{(10)}(\tau ) \end{aligned}$$

and

$$\begin{aligned} H_{(10)}\bigg (-\frac{1}{\tau }\bigg )=\sqrt{-i\tau } \ \frac{2}{\sqrt{5}} \ M_{(10)} \ H_{(10)}(\tau ), \end{aligned}$$

where the matrices $N_{(10)}$ and $M_{(10)}$ are defined as in (3.26) and (3.27).

The following result is a simple consequence from the statement above.

#### Lemma 3.47

The function

$$\begin{aligned} \widetilde{H}_{(10)}:&=(h_{(10),0}+h_{(10),2}) \ [-\mathfrak {e}_6+\mathfrak {e}_{-6}]+(h_{(10),0}-h_{(10),2}) \ [-\mathfrak {e}_4+\mathfrak {e}_{-4}] \\&\quad +(h_{(10),1}+h_{(10),3}) \ [-\mathfrak {e}_2+\mathfrak {e}_{-2}]+(h_{(10),1}-h_{(10),3}) \ [-\mathfrak {e}_8+\mathfrak {e}_{-8}] \\&\quad +h_{(10),4} \ [\mathfrak {e}_1-\mathfrak {e}_{-1}-\mathfrak {e}_9+\mathfrak {e}_{-9}]+h_{(10),5} \ [\mathfrak {e}_3-\mathfrak {e}_{-3}-\mathfrak {e}_7+\mathfrak {e}_{-7}] \end{aligned}$$

transforms with respect to the dual Weil representation $\overline{\rho }_L$ of weight $\nicefrac {1}{2}$ and level $N=10$.

#### Definition 3.48

We define the function

3.28 $$\begin{aligned} e_{(10)}(z):=\bigg (\frac{\eta (z)\eta (2z)}{\eta (5z)\eta (10z)}\bigg )^2-25\bigg (\frac{\eta (5z)\eta (10z)}{\eta (z)\eta (2z)}\bigg )^2. \end{aligned}$$

This function is a weakly holomorphic modular form of weight 0, level 10 whose principal part starts with $q^{-1}$.

#### Theorem 3.49

Let $e_{(10)}(z)\in M_0^!(10)$ be defined as in (3.28).

1. For $n\ge 1$ the coefficients $a_X(n)$ of *X*(*q*) are given by
  $$\begin{aligned} a_X(n)&=\frac{i}{2\sqrt{40n-1}}\big (tr _{e_{(10)}}^+(1-40n,1)-tr _{e_{(10)}}^-(1-40n,1) \big ). \end{aligned}$$
2. For $n\ge 1$ the coefficients $a_\chi (n)$ of $\chi (q)$ are given by
  $$\begin{aligned} a_\chi (n)&=\frac{i}{2\sqrt{40n-9}}\big (tr _{e_{(10)}}^+(9-40n,3)-tr _{e_{(10)}}^-(9-40n,3) \big ). \end{aligned}$$
3. For $n\ge 1$ the coefficients $a_\phi (n)$ of $\phi (q)$ are given by
  $$\begin{aligned} a_\phi (n)&={\left\{ \begin{array}{ll}\frac{-i}{4\sqrt{40\frac{n+2}{2}-36}}\big (tr _{e_{(10)}}^+(36-40\frac{n+2}{2},6)-tr _{e_{(10)}}^-(36-40\frac{n+2}{2},6) \big ),&{}\text { if }n\text { is even,}\\ \frac{-i}{4\sqrt{40\frac{n+1}{2}-16}}\big (tr _{e_{(10)}}^+(16-40\frac{n+1}{2},4)-tr _{e_{(10)}}^-(16-40\frac{n+1}{2},4) \big ),&{}\text { if }n\text { is odd.} \end{array}\right. } \end{aligned}$$
4. For $n\ge 1$ the coefficients $a_\psi (n)$ of $\psi (q)$ are given by
  $$\begin{aligned} a_\psi (n)&={\left\{ \begin{array}{ll}\frac{-i}{4\sqrt{40\frac{n}{2}-4}}\big (tr _{e_{(10)}}^+(4-40\frac{n}{2},2)-tr _{e_{(10)}}^-(4-40\frac{n}{2},2) \big ),&{}\text { if }n\text { is even,}\\ \frac{-i}{4\sqrt{40\frac{n+3}{2}-64}}\big (tr _{e_{(10)}}^+(64-40\frac{n+3}{2},8)-tr _{e_{(10)}}^-(64-40\frac{n+3}{2},8) \big ),&{}\text { if }n\text { is odd.} \end{array}\right. } \end{aligned}$$
